# The Role of ZNF275/AKT Pathway in Carcinogenesis and Cisplatin Chemosensitivity of Cervical Cancer Using Patient-Derived Xenograft Models

**DOI:** 10.3390/cancers15235625

**Published:** 2023-11-28

**Authors:** Miaomiao Ye, Tingxian Liu, Liqing Miao, Shuangwei Zou, Huihui Ji, Jian’an Zhang, Xueqiong Zhu

**Affiliations:** Zhejiang Provincial Clinical Research Center for Obstetrics and Gynecology, Department of Obstetrics and Gynecology, The Second Affiliated Hospital of Wenzhou Medical University, Wenzhou 325027, China; 223012@wzhealth.com (M.Y.);

**Keywords:** cervical cancer, zinc finger protein 275 (ZNF275), cisplatin, chemosensitivity, patient-derived xenograft (PDX) models

## Abstract

**Simple Summary:**

Zinc finger protein 275 (ZNF275) is a C2H2-type transcription factor localized on chromosome Xq28. The present study indicated that cervical cancer tissue exhibited a higher expression of ZNF275 in contrast to the surrounding normal cervical tissue. Moreover, downregulated expression of ZNF275 showed a tumor-inhibitory impact on cervical cancer involving a weakening of the AKT/Bcl-2 signaling pathway. Furthermore, triciribine, targeting and reducing signaling in the ZNF275-induced AKT pathway, exhibited a tumor-suppressive role and sensitized cisplatin chemotherapy efficacy in cervical cancer cells expressing high ZNF275. In addition, the combination treatment of triciribine and cisplatin was more effective in inducing tumor regression than single agents in cervical cancer PDX models expressing high ZNF275. Our findings show that ZNF275 potentially acts as a sufficiently predictive biomarker for the therapeutic effectiveness of the combined administration of triciribine and cisplatin in cervical cancer.

**Abstract:**

Zinc finger protein 275 (ZNF275) is a C2H2-type transcription factor that is localized on chromosome Xq28. Whether ZNF275 participates in modulating the biological behaviors of cervical cancer has not been determined to our knowledge. The present study employed CCK-8, BrdU, flow cytometry, and a transwell assay to investigate the cell viability, proliferation, apoptosis, migration, and invasion of cervical cancer cells. The application of Western blotting and immunohistochemistry (IHC) aims to assess ZNF275 protein expression and identify the signaling pathway relevant to ZNF275-mediated effects on cervical cancer. The therapeutic impact of the combined therapy of the AKT inhibitor triciribine and cisplatin was evaluated on cervical cancer patient-derived xenograft (PDX) models expressing high ZNF275. The current research illustrated that cervical cancer tissue exhibited a higher expression of ZNF275 in contrast to the surrounding normal cervical tissue. The downregulation of ZNF275 suppressed cell viability, migration, and invasion, and facilitated the apoptosis of SiHa and HeLa cells via weakening AKT/Bcl-2 signaling pathway. Moreover, triciribine synergized with cisplatin to reduce cell proliferation, migration, and invasion, and enhanced the apoptosis of SiHa cells expressing high ZNF275. In addition, the combination treatment of triciribine and cisplatin was more effective in inducing tumor regression than single agents in cervical cancer PDX models expressing high ZNF275. Collectively, the current findings demonstrated that ZNF275 serves as a sufficiently predictive indicator of the therapeutic effectiveness of the combined treatment of triciribine and cisplatin on cervical cancer. Combining triciribine with cisplatin greatly broadens the therapeutic options for cervical cancer expressing high ZNF275, but further research is needed to confirm these results.

## 1. Introduction

Cervical cancer is identified as the fourth most frequently occurring malignancy and ranks fourth in cancer-related mortality in females [1]. Cervical cancer patients are primarily treated with surgery or a concurrent chemoradiotherapy regimen [2]. Additionally, chemotherapy is the regimen of choice for cervical cancer with advanced local distribution, metastatic spread, and recurrence, and cisplatin is widely classified as the most effective chemotherapy drug [3,4]. Recent research has shown that somatic mutations and copy number variation (CNV) in cervical cancer potentially promote/inhibit the development or are related to the chemotherapy resistance of cervical cancer [5,6].

Patient-derived xenograft (PDX) models are constructed by engrafting fresh tissues resected during surgery or biopsy into immunodeficient mice, which are increasingly employed to explore tumor evolution and develop personalized therapeutic regimes [7,8]. Our previous study successfully constructed serially transplantable cervical cancer PDX models, which are genetically and histologically similar to patient tissues [9]. Additionally, the mutation analysis identified that members of the zinc-finger protein (ZNF) family, including ZNF275 and ZNF280A, were overlapping mutations in the original tissues and the corresponding PDX tissues [9].

ZNFs are reported as a large family of transcription factors, and previous studies have elucidated eight categories of zinc finger motifs, of which the Cys2-His2 (C2H2)-type zinc finger motif is one of the most frequent binding regions for DNA [10,11]. Different ZNFs exert distinctive functional interactions in biological processes of various cancers, which are involved in tumorigenesis, cancer progression, and metastasis [12,13,14]. ZNF275 is a C2H2-type transcription factor localized on chromosome Xq28 [15]. One study performed a weighted gene co-expression network analysis to figure out that ZNF275 had the potential to differentiate the naive and primed states of pluripotency in human, which had not previously been reported and could be of interest for further validation experiments [16]. In addition, the specific roles of ZNF275 in cervical cancer as well as other cancers have not been systematically addressed yet.

The AKT signaling pathway is dysregulated in some cancers and is involved in carcinogenesis and cancer development [17,18]. Therefore, AKT is considered as a validated target for developing cancer therapeutic modalities, and triciribine has been shown to function in the pharmacological inhibition of AKT activation [19,20]. Liu et al. [21] illustrated that phosphorylation of AKT was induced in pancreatic cancer cells with the depletion of HEAT repeat-containing protein 1 (HEATR1). This research also demonstrated that treatment of the AKT inhibitor triciribine improved the chemosensitivity of gemcitabine in HEATR1-depleted pancreatic cancer cells [21]. One study determined that ZNF217 mediated collagen matrix abundance-induced AKT activation and mammary epithelial cell proliferation, and triciribine could potentially be utilized as a chemo-preventive agent to decrease the risk of breast cancer for females identified as having enhanced mammographic density [22].

This study investigated ZNF275 expression and its functional effects and molecular mechanisms in cervical cancer. ZNF275 was down-regulated in SiHa and HeLa cells to evaluate its impact on the proliferative, apoptotic, migrative, and invasive abilities of cervical cancer. Moreover, whether the AKT signaling pathway exerted functions in ZNF275 downregulation-mediated influences in cervical cancer was evaluated. Furthermore, the proliferation, apoptosis, migration, and invasion behaviors of SiHa and Caski cells following exposure to the combination of triciribine and cisplatin were explored. Additionally, the established cervical cancer PDX models were utilized to observe the efficiency of the co-treatment of triciribine and cisplatin in vivo. The present research proposes that the combination treatment of triciribine and cisplatin potentially broadens the therapeutic modality for cervical cancer expressing high ZNF275.

## 2. Materials and Methods

### 2.1. Clinical Tissue Samples

Five fresh cervical cancer samples and paired surrounding normal cervical samples were randomly obtained from patients who were pathologically and clinically diagnosed with cervical squamous cell carcinoma and treated with surgical excision at the Second Affiliated Hospital of Wenzhou Medical University. These patients did not undergo chemo- or radio-therapy prior to surgery. The fresh resected samples were stored in liquid nitrogen before protein extraction and Western blotting.

### 2.2. Cell lines and Culture

The cervical cancer cell lines SiHa, HeLa, MS751, and Caski were obtained from the Shanghai Cell Biology Medical Research Institute, Chinese Academy of Sciences. The human immortalized normal cervical epithelial cell line ECT1/E6E7 was obtained from Shanghai BinSui Biological Technology Co., Ltd. (Shanghai, China). SiHa, HeLa, and MS751 cells were incubated in Dulbecco’s modified Eagle’s medium (DMEM), Caski was incubated in Roswell Park Memorial Institute (RPMI)-1640, and ECT1/E6E7 was incubated in Eagle’s minimum essential medium (EMEM), with a supplement of 10% fetal bovine serum (FBS) and 1% penicillin–streptomycin solution (100 mg/mL). Cells were maintained at 37 °C in a humid environment containing 5% CO_2_.

### 2.3. Compounds

The AKT activator SC79 (#HY-18749; MedChemExpress (MCE), NJ, USA) and triciribine (#HY-15457; MCE, NJ, USA) were solubilized in dimethyl sulfoxide (DMSO) to prepare a stock solution of 100 mM and 200 mM, respectively. The cisplatin (#HY-17394; MCE, NJ, USA) was solubilized in distilled water at a concentration of 2 mM. The stock solutions of SC79, triciribine, and cisplatin were cryopreserved at −20 °C, and the corresponding culture medium was utilized for dilution to specific concentrations prior to usage.

### 2.4. Database Mining

The Gene Expression Omnibus (GEO) database (https://www.ncbi.nlm.nih.gov/geo/ (accessed on 18 August 2023.)) [23] was used to explore the expression of ZNF275 in cervical cancer tissues and normal cervical tissues. The expression association of ZNF275 and AKT in cervical cancer samples was identified via the Gene Expression Profiling Interactive Analysis (GEPIA) database (http://gepia.cancer-pku.cn/ (accessed on 9 February 2022)) [24], using Spearman’s correlation coefficient analysis.

### 2.5. Lentivirus and Transfection

SiHa, HeLa, and ECT1/E6E7 cells were infected with lentivirus encoding ZNF275 shRNAs. The ZNF275 shRNAs and control plasmids were obtained from Shanghai Jiao Tong University School of Medicine. The sequences of the shRNAs were as follows. ZNF275-shRNA-1: CCCATTGAATGCAGCATTA and ZNF275-shRNA-2: GTATTCCTGTTGTGAGGAA. Lentiviruses were packaged in 293T cells using packaging vectors psPAX2 and pMD2.G with the addition of lipofectamine 2000. The recombinant lentiviral particles in cell culture supernatants were collected and filtered for further usage. To stably downregulate ZNF275 expression, the recombinant lentiviral particles (pLKO.1-TRC-shRNA-1, pLKO.1-TRC-shRNA-2, and its control shNC) were generated to transduce cells with the addition of polybrene (8 µg/mL). Transfected cells (ZNF275 shRNA and control) were placed into selection medium supplemented with 2 μg/mL puromycin for 48 h. Subsequently, the knockdown efficiency of ZNF275 in cells was validated by Western blotting.

### 2.6. Colony Formation Assay

Transfected SiHa and HeLa cells (ZNF275 shRNA and control) were inoculated in 6-well plates (1 × 10^3^ cells/well), and then cultured for two weeks to allow for the formation of macroscopically visible colonies. Afterwards, cell colonies were fixed with 4% paraformaldehyde for 15 min, and stained with crystal violet for 15 min, and then quantified under a microscope.

### 2.7. Cell Viability Assay

Cell counting kit-8 (CCK-8) assay was applied to assess cell viability. Transfected cells (ZNF275 shRNA and control) were inoculated in 96-well plates (2 × 10^3^ cells/well), and then cultured for 24 h, 48 h, and 72 h. Afterward, the culture medium was discarded and replaced with 100 μL serum-free DMEM containing 10 μL CCK-8 solution. Then, the absorbance at 450 nm was read with a Microplate Reader (Bio-Rad, Hercules, CA, USA).

Additionally, SiHa cells (6 × 10^3^ cells/well) and Caski cells (4 × 10^3^ cells/well) were inoculated in 96-well plates. Cells were next exposed to different concentrations of triciribine for 24 h, 48 h and 72 h. Moreover, different concentrations of cisplatin were administered to cells for 48 h. Afterward, cells were co-incubated with triciribine and cisplatin for 48 h. Subsequently, cell viability was evaluated by CCK-8 assay as mentioned above.

### 2.8. Drug Combination Index

Combination index (CI) was quantified to evaluate the efficacy of combined drug treatment in suppressing cell proliferation by ComboSyn software version 3.0.1. The combined effect can be defined as synergism (CI < 1), additivity (CI = 1), and antagonism (CI > 1) [25]; the smaller the CI value, the stronger the synergy [26].

### 2.9. Transwell Migration and Invasion Assays

Polycarbonate transwell inserts with 8 μm pores were applied to assess the migrative and invasive capacities of cervical cancer cells (SiHa and HeLa). Transfected cells (ZNF275 shRNA and control) with a density of 3 × 10^4^ cells/well for migration and 6 × 10^4^ cells/well for invasion in serum-free DMEM were inoculated in the upper compartment. The bottom compartment was filled with 500 μL of DMEM plus 10% FBS. For the invasion assay, the upper compartments of transwell inserts were pretreated with 100 μL Matrigel matrix diluted at a ratio of 1:4 in serum-free DMEM. Approximately 24 h later, transwell inserts were carefully washed twice with PBS. Afterward, cells migrating or invading across the membrane were fixed with 4% paraformaldehyde for 20 min and stained with 1% crystal violet for 10 min. The migrated or invaded cells were visualized and quantified under a microscope (Leica Microsystems, Wetzlar, Germany).

Furthermore, SiHa and Caski cells were pretreated with different reagents (triciribine or cisplatin, or a combination of triciribine and cisplatin) for 48 h. After that, the negative control cells and these different pretreated cells were inoculated in the upper compartments for migration (3 × 10^4^ cells/well for SiHa; 5 × 10^4^ cells/well for Caski) and invasion (1.6 × 10^5^ cells/well for SiHa; 1.0 × 10^5^ cells/well for Caski) in serum-free DMEM. The next experimental steps were carried out in the same way as described above.

### 2.10. Flow Cytometric Analysis

Cell apoptosis was evaluated with AnnexinV-PE or Annexin V-FITC Apoptosis Detection kits. Transfected cells (ZNF275 shRNA and control) were inoculated in a 60-mm dish (5 × 10^5^ cells) and incubated for 24 h. Afterward, cells were gently trypsinized, and cell suspensions were prepared using binding buffer. Subsequently, cell apoptosis was assessed after staining with 5 µL PE Annexin V and 5 µL 7-AAD by CytoFLEX flow cytometry (Beckman Coulter, Fullerton, CA, USA).

Additionally, SiHa and Caski cells were pretreated with different reagents (triciribine, or cisplatin, or a combination of triciribine and cisplatin) for 48 h. Negative control cells were maintained in complete DMEM for 48 h. Finally, apoptosis of SiHa cell was assessed after staining with 5 µL PE Annexin V and 5 µL 7-AAD, and apoptosis of Caski cell was assessed after staining with 5 μL FITC Annexin V and 5 μL PI.

### 2.11. Bromodeoxyuridine (BrdU) Proliferation Assay

The BrdU incorporation assay was employed to evaluate cell proliferation. Cells were inoculated in 6-well plates (3 × 10^5^ cells/well for SiHa; 2 × 10^5^ cells/well for Caski) on sterilized coverslips. Adherent cells were administered with different reagents (triciribine, or cisplatin, or a combination of triciribine and cisplatin) for 48 h. Afterward, BrdU was added to negative control cells and these different treated cells at a 30 μg/mL final concentration 6 h prior to culture termination. After fixing with 4% paraformaldehyde for 30 min, cells were incubated in 0.2% Triton X-100 for 20 min for permeabilization, and maintained in blocking buffer (3% bovine serum albumin) for 1 h. Subsequently, the primary anti-BrdU mouse monoclonal antibody (1:200, #5292; CST) was employed to label cells at 4 °C overnight, followed by secondary antibody incubation for 1 h at room temperature and shielding from light. Ultimately, positive staining of cells was performed with 3,3′-diaminobenzidine (DAB) for 2 min, and counterstaining of nuclei was performed with hematoxylin for 20 s. The BrdU-positive cells, which were supposed to be in the proliferative phase, were visualized and quantified under the microscope (Leica Microsystems, Wetzlar, Germany).

### 2.12. Western Blotting Analysis

Radio immunoprecipitation assay (RIPA) lysis buffer with the addition of phenylmethanesulfonylfluoride (PMSF) was employed for collecting cell lysates. Bicinchoninic Acid (BCA) Protein Assay kit was utilized for protein concentration quantification. Protein aliquots were electrophoresed with sodium dodecyl sulfate PAGE and electroblotted onto polyvinylidene difluoride (PVDF) membrane, followed by overnight incubation of primary antibodies at 4 °C with slight shaking. The primary antibodies against ZNF275 (1:1000, #HPA000566; Atlas Antibodies), AKT (1:1000, #4685; CST), p-AKT (1:1000, #4060; CST), Bcl-2 (1:1000, #4223; CST), Bax (1:1000, #5023; CST), and GAPDH (1:3000, 60004-1-Ig; Proteintech) were applied. After that, membranes were hybridized with horseradish peroxidase-conjugated secondary antibody for 90 min at room temperature. Ultimately, a chemiluminescence imaging system (ChemiScope 6000, CLiNX, Huangshan, China) was used to scan the protein bands, and Image J software version 1.42q. was used for Western blotting quantification.

### 2.13. Establishment of Cervical Cancer PDX Models

Female NOD-SCID mice aged 4–6 weeks were procured from Beijing Vital River Laboratory Animal Technology of China and housed in isolator cages with filter covers at Wenzhou Medical University in a specific-pathogen-free (SPF) barrier facility. For the establishment of cervical cancer PDX models, small pieces of approximately 3 mm^3^ of fresh cervical cancer specimens (termed as F0 generation), which were obtained instantly following surgical excision in the operating room, were inoculated subcutaneously on the right buttock of female NOD-SCID mice aged 6–8 weeks. The mice that were transplanted with patient-originated cervical cancer tissues were considered the F1 generation, and the following generations were sequentially numbered as F2, F3…Fn. Mice underwent euthanasia when the tumor reached 1000 mm^3^ in volume. Tumors were resected and dissociated for serial passaging of cervical cancer PDX models and preparation of formalin-fixed paraffin-embedded tissue samples. The F3-PDX mice were further utilized for in vivo drug treatment studies.

### 2.14. Immunohistochemistry (IHC) Staining

The patient original cervical cancer tissue (F0), paired adjacent non-cancerous cervical tissue (N0), and F2- and F3-PDX tissues were obtained for IHC. Following fixation with 4% paraformaldehyde and paraffin embedding, tissues were cross-sectionally sliced as 4 μm thick sections. Antigen retrieval with the use of a microwave oven was administered to tissue sections, followed by processing with primary antibody against ZNF275 (1:200) overnight at 4 °C. Then, tissue slides were hybridized with secondary antibody at room temperature for 90 min. After that, antigen visualization in tissue sections was performed with DAB solution for 2 min, and the cell nuclei were counterstained with hematoxylin for 20 s. The presence of yellowish-brown staining in nuclei was considered positive. A microscope (Leica Microsystems, Wetzlar, Germany) was used to acquire the representative pictures of tissues.

### 2.15. In Vivo Drug Treatment Study

When the tumors reached 100 mm^3^ in volume, F3-PDX mice were assigned at random to four clusters consisting of four mice in each cluster. F3-PDX mice were intraperitoneally injected with either PBS (control), triciribine (12 mg/kg), cisplatin (4 mg/kg), or a combination of triciribine (12 mg/kg) and cisplatin (4 mg/kg) every 5 days with 4 injections in total. A Vernier caliper was utilized to monitor the length and width of the tumors every 5 days, and tumor volume was computed with the following formula [27]: Volume (mm^3^) = length × width^2^/2. On day 5 after the final treatment, mice were euthanized by cervical dislocation, and xenograft tumors were subsequently separated and photographed with a camera.

Subsequently, hematoxylin–eosin (HE) staining was conducted for pathological analysis of tumor tissues. Protein expression of ZNF275, AKT, and p-AKT was evaluated in tumor tissues using IHC staining.

### 2.16. Statistical Analysis

The Kolmogorov–Smirnov test was conducted to check the normality of variables. When statistical data exhibited a normal distribution, data were expressed as mean ± standard deviation (SD), and Student’s *t*-test (for two-group comparisons) or ANOVA (for multiple-group comparisons) was employed for differential analysis. When statistical data presented a non-normal distribution, data were expressed as median (interquartile range), and a Mann–Whitney test was chosen for differential analysis. Experimental data were statistically analyzed in GraphPad Prism 8.0 (GraphPad Software, San Diego, CA, USA), and figures were graphed with mean ± SD. *p* < 0.05 was deemed statistically significant (* *p* < 0.05, ** *p* < 0.01, *** *p* < 0.001, **** *p* < 0.0001).

## 3. Results

### 3.1. Expression Level of ZNF275 Protein in Cervical Cancer

**Figure 1:**
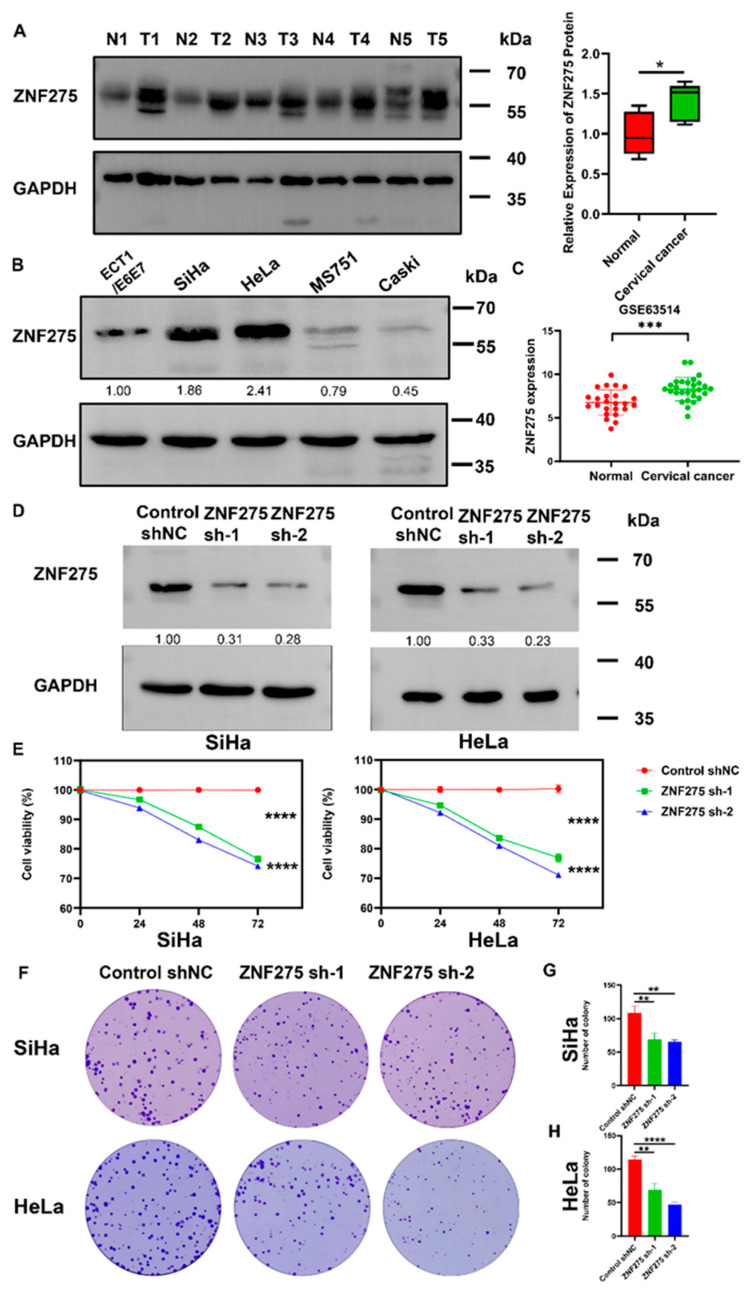
The effect of ZNF275 knockdown on viability of cervical cancer cells. (**A**) Left panel: ZNF275 protein expression in cervical cancer tissues and adjacent normal tissues was detected by Western blotting. N: normal tissue. T: cervical cancer tissue. Right panel: Quantitative analysis of the results. (**B**) The expression of ZNF275 protein in cervical cancer cell lines was determined by Western blotting. (**C**) The differential expression of ZNF275 in cervical cancer tissues and normal cervical tissues was retrieved in Gene Expression Omnibus (GEO) database. (**D**) The efficiencies of ZNF275 knockdown in SiHa and HeLa cells were validated by Western blotting. (**E**) CCK-8 assay revealed the effect of ZNF275 downregulation on viabilities of SiHa and HeLa cells. (**F**) Colony formation assay revealed the effect of ZNF275 downregulation on clonogenicity of SiHa and HeLa cells. (**G**) Quantitative analysis of the results in (**F**) of SiHa cells. (**H**) Quantitative analysis of the results in (**F**) of HeLa cells. In all panels, * *p* < 0.05, ** *p* < 0.01, *** *p* < 0.001, **** *p* < 0.0001 vs. control or normal groups. The uncropped Western blots are shown in File S1.

ZNF275 protein was highly expressed in cervical cancer specimens when compared with surrounding normal tissues (Figure 1A). In comparison to ECT1/E6E7 cells, ZNF275 protein expression was significantly increased in SiHa and HeLa cells (Figure 1B). Moreover, the GSE63514 dataset from the GEO database displayed that ZNF275 was more expressed in cervical cancer tissues than normal cervical tissues (Figure 1C).

Moreover, SiHa and HeLa cells were selected to establish the stable ZNF275-knockdown cell models. Downregulation of ZNF275 in SiHa and HeLa cells was observed with both ZNF275 sh-1 and ZNF275 sh-2 transfections by Western blotting analysis (Figure 1D). Furthermore, in contrast to their control groups, the viabilities of SiHa cells with ZNF275 knockdown (ZNF275-KD-SiHa) and HeLa cells with ZNF275 knockdown (ZNF275-KD-HeLa) were evidently suppressed (Figure 1E). Consistently, in comparison with their control groups, ZNF275 knockdown decreased the colony formation of SiHa and HeLa cells (Figure 1F–H).

The present study additionally explored the effect of ZNF275 downregulation on ECT1/E6E7 cells. Both ZNF275 sh-1 and ZNF275 sh-2 transfections downregulated the expression of ZNF275 protein in ECT1/E6E7 cells (Supplementary Figure S1A). Nevertheless, ZNF275 knockdown exhibited no observable significant impact on cell viabilities of ECT1/E6E7 (Supplementary Figure S1B). Consistently, downregulation of ZNF275 showed no effect on apoptosis of ECT1/E6E7 cells (Supplementary Figure S1C,D).

### 3.2. Effects of ZNF275 Downregulation on Migrative and Invasive Abilities of Cervical Cancer Cells

**Figure 2:**
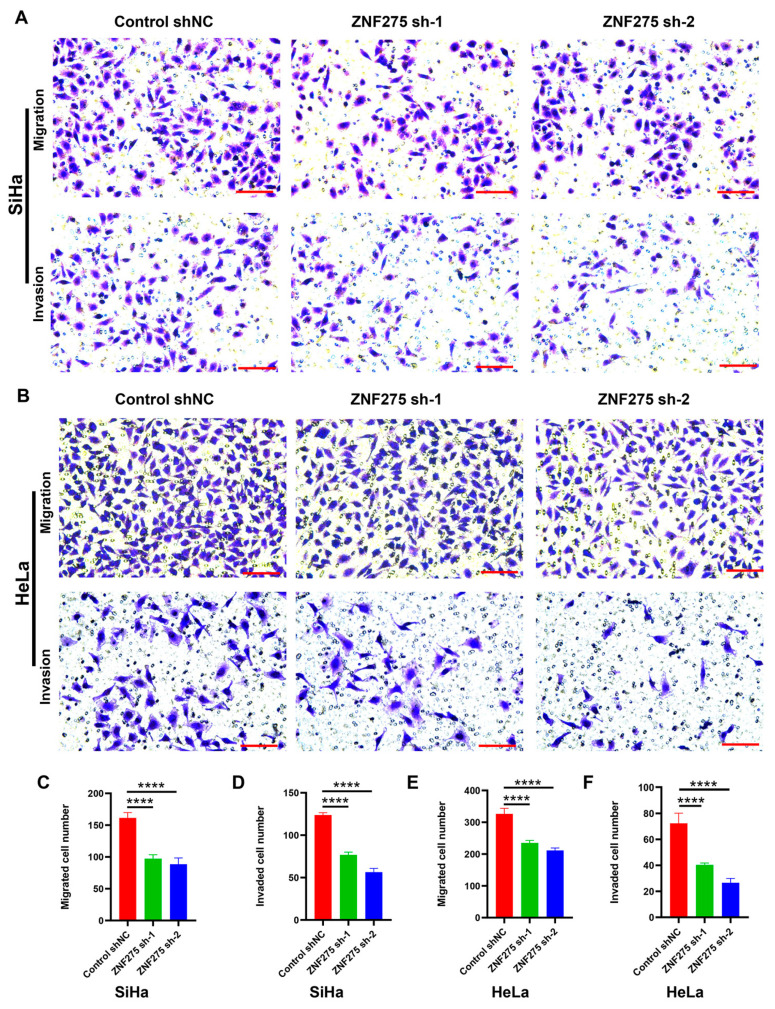
The effect of ZNF275 knockdown on migration and invasion of cervical cancer cells. (**A**,**B**) Transwell assay revealed the effect of ZNF275 downregulation on migration and invasion of SiHa (**A**) and HeLa (**B**) cells in comparison of their control groups. (**C**,**D**) Quantitative analysis of the results of migration (**C**) and invasion (**D**) in (**A**). (**E**,**F**) Quantitative analysis of the results of migration (**E**) and invasion (**F**) in (**B**). Scale bar = 100 µm. **** *p* < 0.0001 vs. control groups.

The migrative (Figure 2A,C for SiHa; Figure 2B,E for HeLa) and invasive (Figure 2A,D for SiHa; Figure 2B,F for HeLa) capacities were attenuated in ZNF275-KD-SiHa and ZNF275-KD-HeLa cells, when compared to their corresponding control groups.

### 3.3. Effects of ZNF275 Downregulation on Apoptosis of Cervical Cancer Cells

**Figure 3:**
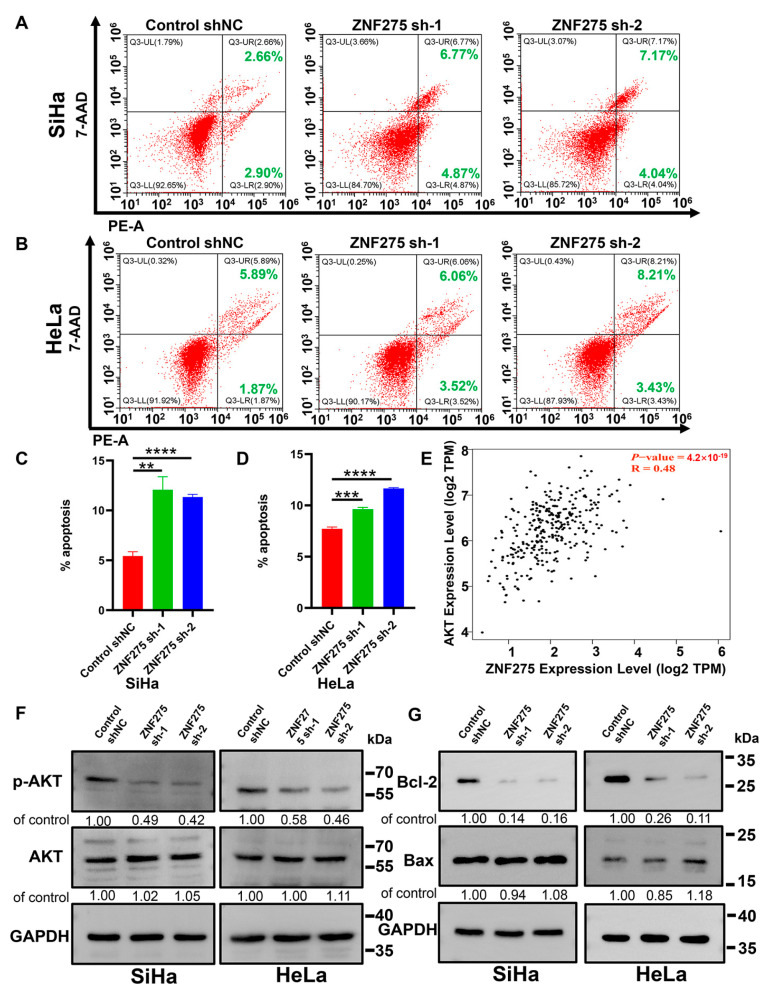
The effect of ZNF275 knockdown on apoptosis of cervical cancer cell. (**A**,**B**) Flow cytometric analysis revealed the effect of ZNF275 downregulation on apoptosis of SiHa (**A**) and HeLa (**B**) cells in comparison of their control groups. (**C**) Quantitative analysis of the results of (**A**). (**D**) Quantitative analysis of the results of (**B**). ** *p* < 0.01, *** *p* < 0.001, **** *p* < 0.0001 vs. control groups. (**E**): The relationship between ZNF275 and AKT expression in cervical cancer samples was explored in the Gene Expression Profiling Interactive Analysis (GEPIA) database. (**F**) The expression levels of AKT and p-AKT protein in ZNF275 knockdown SiHa and HeLa cells in comparison to their control groups were detected by Western blotting. (**G**) The expression levels of apoptosis-associated protein Bcl-2 and Bax in ZNF275 knockdown SiHa and HeLa cells in comparison to their control groups were detected by Western blotting. The uncropped Western blots are shown in File S1.

The apoptosis of ZNF275-KD-SiHa and ZNF275-KD-HeLa cells was enhanced compared to their corresponding control groups (Figure 3A,C for SiHa; Figure 3B,D for HeLa). Furthermore, a positive correlation between ZNF275 and AKT expression was revealed in the GEPIA database (Figure 3E). Western blotting was chosen to further validate the correlation between ZNF275 and AKT pathway. Knockdown of ZNF275 decreased phosphorylated-AKT (p-AKT) levels in SiHa and HeLa cells, versus their respective control groups (Figure 3F). Additionally, Western blotting analysis uncovered that the level of apoptosis-associated protein Bcl-2 was downregulated while Bax protein expression remained unchanged, showing an increasing tendency of Bax/Bcl-2 ratio in ZNF275-KD-SiHa and ZNF275-KD-HeLa cells compared to that in their control groups (Figure 3G).

### 3.4. Activation of AKT/Bcl-2 Signaling Pathway Rescued ZNF275 Downregulation Mediated Effects on Cervical Cancer Cell Viability and Apoptosis

**Figure 4:**
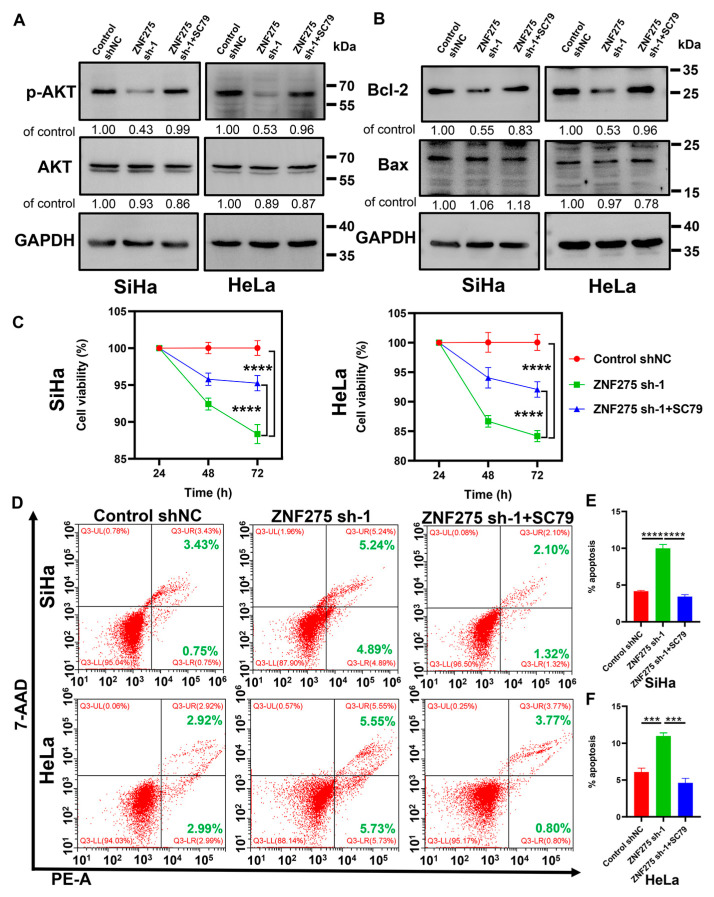
The effect of AKT activation by treating with 10 μM SC79 for 48 h on ZNF275 knockdown cervical cancer cells. (**A**) The expression levels of AKT and p-AKT protein were detected by Western blotting. (**B**) The expression levels of Bcl-2 and Bax protein were detected by Western blotting. (**C**) Left panel: The cell viability of SiHa cells was revealed by CCK-8 assay. Right panel: The cell viability of HeLa cells was revealed by CCK-8 assay. (**D**) The apoptosis of SiHa and HeLa cells was analyzed by flow cytometry. (**E**) Quantitative analysis of the results of SiHa cells in (**D**). (**F**) Quantitative analysis of the results of HeLa cells in (**D**). *** *p* < 0.001, **** *p* < 0.0001 vs. control groups or ZNF275 sh-1 groups. The uncropped Western blots are shown in File S1.

To further demonstrate the role of the AKT signaling pathway in ZNF275 knockdown cervical cancer cells, reversal experiments with the treatment of 10 μM SC79 for 48 h in cervical cancer cells were employed. Expression of p-AKT (Figure 4A) and Bcl-2 (Figure 4B) protein was upregulated in ZNF275-KD-SiHa and ZNF275-KD-HeLa cells with the treatment of 10 μM SC79 for 48 h. Moreover, 10 μM SC79-induced AKT activation rescued the inhibition of cell viabilities in ZNF275-KD-SiHa and ZNF275-KD-HeLa cells (Figure 4C). Additionally, AKT activation mediated by 10 μM SC79 reversed the promotion of apoptosis in ZNF275-KD-SiHa and ZNF275-KD-HeLa cells (Figure 4D–F).

### 3.5. Therapeutically Targeting ZNF275-Induced AKT Pathway with Triciribine Influenced the Viabilities of SiHa and Caski Cells

**Figure 5:**
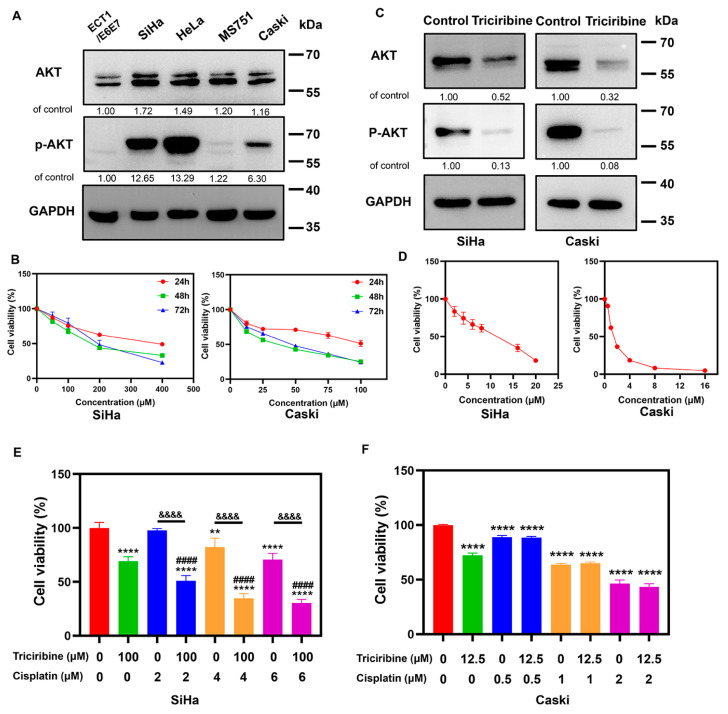
The effect of triciribine and cisplatin on cell viability of SiHa and Caski cells. (**A**) The expression of AKT and p-AKT protein in cervical cancer cell lines were determined by Western blotting. (**B**) Left panel: The viabilities of SiHa cells at different concentrations of triciribine (0, 50, 100, 200, and 400 μM) for 24 h, 48 h, and 72 h were determined by CCK-8 assay. Right panel: The viabilities of Caski cells at different concentrations of triciribine (0, 12.5, 25, 50, 75, and 100 μM) for 24 h, 48 h, and 72 h were determined by CCK-8 assay. (**C**) Left panel: The effects of triciribine (100 μM for 48 h) on AKT and p-AKT protein expression in SiHa cells were detected by Western blotting. Right panel: The effects of triciribine (12.5 μM for 48 h) on AKT and p-AKT protein expression in Caski cells were detected by Western blotting. (**D**) Left panel: The viabilities of SiHa cells at different concentrations of cisplatin (0, 2, 4, 6, 8, 16, and 20 μM) for 48 h were determined by CCK-8 assay. Right panel: The viabilities of Caski cells at different concentrations of cisplatin (0, 0.5, 1, 2, 4, 8, and 16 μM) for 48 h were determined by CCK-8 assay. (**E**) The viabilities of SiHa cells with the combined treatment of triciribine (100 μM) and different concentrations of cisplatin (0, 2, 4, and 6 μM) for 48 h were determined by CCK-8 assay. (**F**) The viabilities of Caski cells with the combined treatment of triciribine (12.5 μM) and different concentrations of cisplatin (0, 0.5, 1, and 2 μM) for 48 h were determined by CCK-8 assay. ** *p* < 0.01 vs. control group, **** *p* < 0.0001 vs. control group, ^####^ *p* < 0.0001 vs. triciribine group. ^&&&&^ *p* < 0.0001 vs. cisplatin group. The uncropped Western blots are shown in File S1.

Among different cervical cancer cell lines, the AKT and p-AKT protein expression were relatively higher in SiHa cells and relatively lower in Caski cells (Figure 5A), which showed a consistent trend with that of ZNF275 protein expression. Therefore, the present study evaluated the effect of triciribine and cisplatin on SiHa and Caski cells.

CCK-8 assay showed that triciribine reduced the viabilities of SiHa and Caski cells, and treatment with triciribine at 100 μM for 48 h on SiHa and 12.5 μM for 48 h on Caski was identified as the optimal concentration and time point for follow-up experiments (Figure 5B). The AKT and p-AKT protein expression in SiHa and Caski cells was decreased after treating with 100 μM triciribine and 12.5 μM triciribine for 48 h, respectively (Figure 5C).

Moreover, the viabilities of SiHa and Caski cells were inhibited with the treatment of cisplatin for 48 h (Figure 5D). Combined treatment of triciribine (100 μM) and cisplatin (2, 4, and 6 μM) for 48 h, respectively, showed synergistic inhibition of the viabilities of SiHa cells (CI, 0.76, 0.56, and 0.59, respectively) (Figure 5E). Therefore, 100 μM triciribine and 4 μM cisplatin were identified for combination regimen (CI = 0.56) in SiHa cells.

However, the co-administration of triciribine (12.5 μM) and cisplatin (0.5, 1, and 2 μM) in Caski cells for 48 h did not contribute to synergistic repression of cell viabilities (Figure 5F). Ultimately, for Caski cells, the combination treatment of 12.5 μM triciribine (IC 30 value) and 1 μM cisplatin (IC 30 value) for 48 h was selected for the follow-up experiments.

### 3.6. Effects of Triciribine and Cisplatin on Biological Behaviors of SiHa Cells

**Figure 6:**
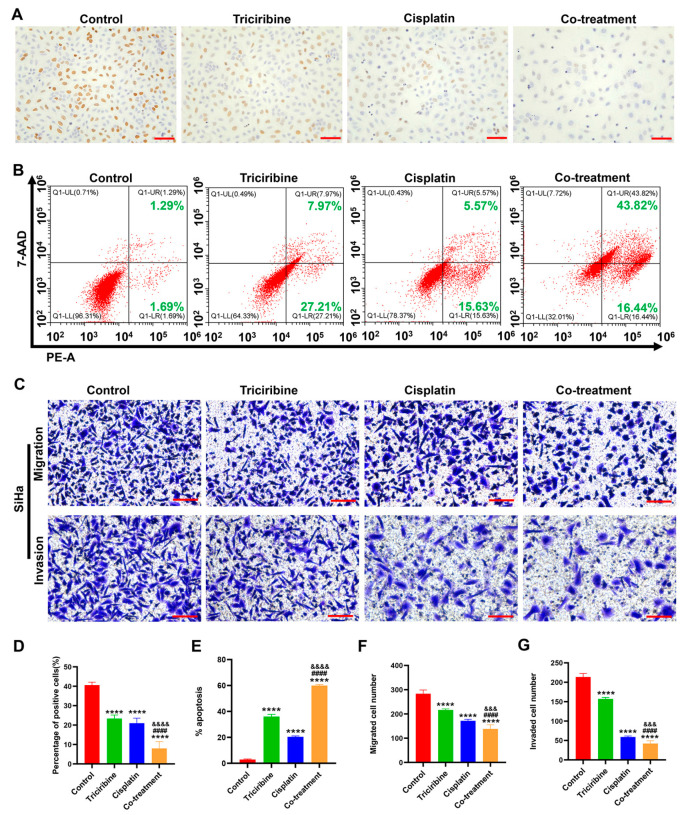
The effect of triciribine combined with cisplatin on the biological behaviors of SiHa cells. SiHa cells were treated with control (complete DMEM), triciribine (100 μM), cisplatin (4 μM), or the co-treatment of triciribine (100 μM) and cisplatin (4 μM) for 48 h. (**A**) The proliferation of SiHa cells was revealed by BrdU assay. Scale bar = 75 µm. (**B**) The apoptosis of SiHa cells was analyzed by flow cytometry (**C**): Transwell assay revealed the migration and invasion of SiHa cells. Scale bar = 100 µm. (**D**) Quantitative analysis of the results of proliferation rates in (**A**). (**E**) Quantitative analysis of the results of apoptosis in (**B**). (**F**,**G**) Quantitative analysis of the results of migration (**F**) and invasion (**G**) in (**C**). **** *p* < 0.0001 vs. control group, ^####^ *p* < 0.0001 vs. triciribine group, ^&&&^ *p* < 0.001 and ^&&&&^ *p* < 0.0001 vs. cisplatin group.

SiHa cells were treated with DMEM (control), triciribine (100 μM), cisplatin (4 μM), or the combination of triciribine (100 μM) and cisplatin (4 μM) for 48 h. In contrast to the control group, the proliferative abilities of SiHa cells treated with single-agent triciribine or single-agent cisplatin were attenuated, while those treated with the combination regimen displayed the most potent inhibition capacity (Figure 6A,D). Compared to the control group, apoptosis of SiHa cells was elevated with the treatment of single-agent triciribine or single-agent cisplatin, while co-treatment with two drugs enhanced this effect (Figure 6B,E). Administration of single-agent triciribine or single-agent cisplatin weakened the migrative and invasive capacities of SiHa cells with respect to the control group (Figure 6C,F,G). Additionally, the combination therapy of triciribine and cisplatin had the potential to successfully weaken the migrative and invasive abilities more effectively than single-agent triciribine or single-agent cisplatin on SiHa cells (Figure 6C,F,G).

### 3.7. Effects of Triciribine and Cisplatin on Biological Behaviors of Caski Cells

**Figure 7:**
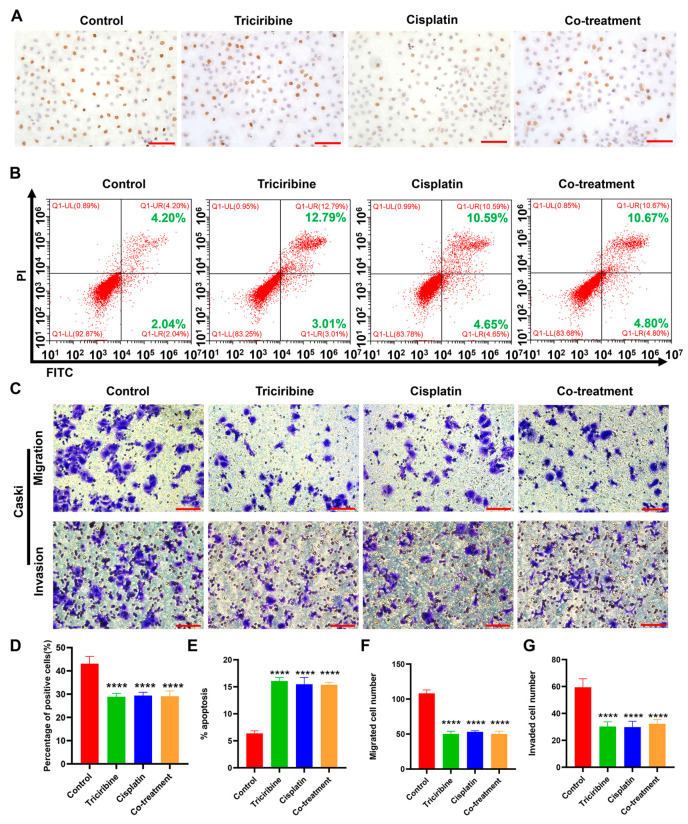
The effect of triciribine combined with cisplatin on the biological behaviors of Caski cells. Caski cells were treated with control (complete DMEM), triciribine (12.5 μM), cisplatin (1 μM), or the combination of triciribine (12.5 μM) and cisplatin (1 μM) for 48 h. (**A**) The proliferation of Caski cells was revealed by BrdU assay. (**B**) The apoptosis of Caski cells was analyzed by flow cytometry. (**C**) Transwell assay revealed the migration and invasion of Caski cells. (**D**) Quantitative analysis of the results of proliferation rates in (**A**). (**E**) Quantitative analysis of the results of apoptosis in (**B**). (**F**,**G**) Quantitative analysis of the results of migration (**F**) and invasion (**G**) in (**C**). Scale bar = 100 µm. **** *p* < 0.0001 vs. the control group.

Caski cells were treated with DMEM (control), triciribine (12.5 μM), cisplatin (1 μM), or the co-treatment of triciribine (12.5 μM) and cisplatin (1 μM) for 48 h. In comparison to the control group, proliferation of Caski cells was reduced when administered with single-agent triciribine or single-agent cisplatin (Figure 7A,D). However, Caski cells did not exhibit a synergistic anti-proliferative response when co-treated with triciribine and cisplatin (Figure 7A,D). Relative to the control group, apoptosis of Caski cells was enhanced with the treatment of single-agent triciribine or single-agent cisplatin, but the combination of triciribine and cisplatin did not further promote apoptosis of Caski cells (Figure 7B,E). In contrast to the control group, the treatment of single-agent triciribine or single-agent cisplatin weakened the migrative and invasive abilities of Caski cells, but the co-administration of triciribine and cisplatin did not induce synergistic effects on migration and invasion of Caski cells (Figure 7C,F,G).

### 3.8. Therapeutic Efficacies of Cisplatin, Triciribine, and the Co-Treatment in Cervical Cancer F3-PDX Mice

**Figure 8:**
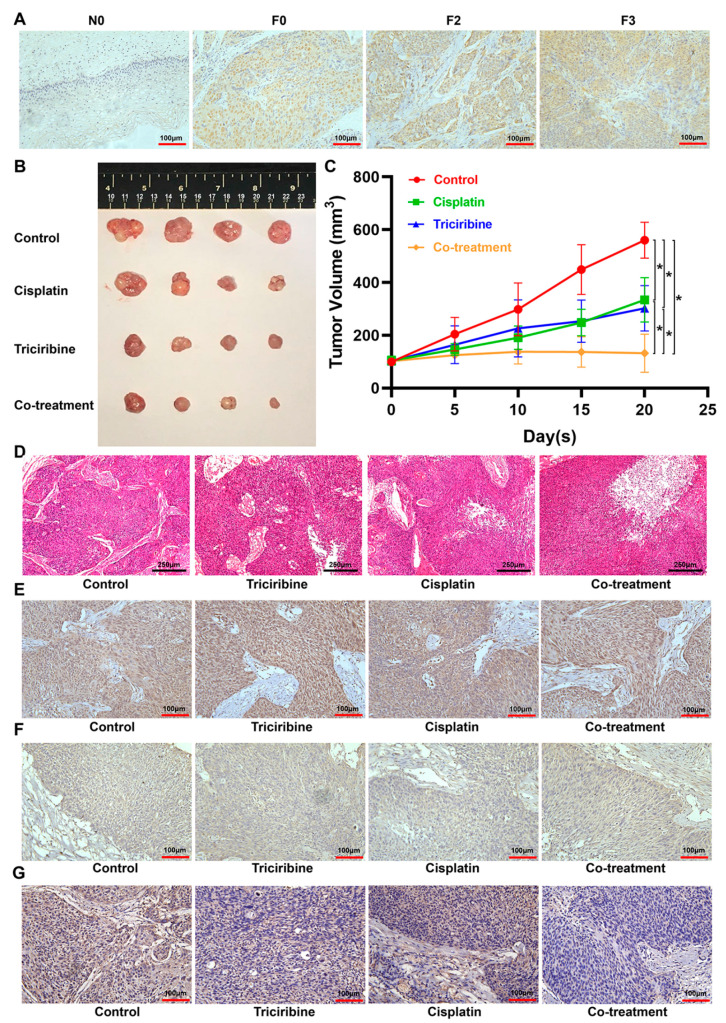
The therapeutic efficacies of cisplatin, triciribine, and the co-treatment were evaluated in cervical cancer F3-PDX mice. Cervical cancer F3-PDX mice were treated with PBS (control), cisplatin (4 mg/kg), triciribine (12 mg/kg), and the co-treatment of cisplatin (4 mg/kg) and triciribine (12 mg/kg). (**A**) Immunohistochemistry staining revealed the protein expression of ZNF275 in patient original cervical cancer tissue (F0), paired adjacent non-cancerous cervical tissue (N0), and F2- and F3-PDX tissue (original magnification, 200×). (**B**) Cervical cancer xenografts were separated and photographed on the 20th day after drug treatment. (**C**) Tumor volumes were measured and calculated. (**D**) The pathology of tumor tissue was observed by hematoxylin–eosin (HE) staining (original magnification, 100×). Immunohistochemistry staining described the protein expression of ZNF275 (**E**), AKT (**F**), and p-AKT (**G**) in tumor tissues with different treatments (original magnification, 200×). * *p* < 0.05 vs. control group or triciribine group or cisplatin group.

In contrast to N0, ZNF275 protein was highly expressed in F0 tissue, which was retained in F2- and F3-PDX tissue (Figure 8A). The therapeutic efficacies of cisplatin (4 mg/kg), triciribine (12 mg/kg), and the co-treatment (the combination of 12 mg/kg triciribine and 4 mg/kg cisplatin) were determined in vivo using the F3-PDX mice. The present study demonstrated that both cisplatin and triciribine suppressed the growth of cervical cancer in F3-PDX mice, in contrast to the control group (Figure 8B,C). Moreover, the inhibitory differences between cisplatin and triciribine in cervical cancer growth showed no statistical significance, indicating that the therapeutic efficacy of triciribine in cervical cancer was at least comparable to cisplatin (Figure 8B,C). Furthermore, the co-treatment with triciribine and cisplatin was more effective in suppressing the growth of cervical cancer than the single-agent cisplatin or single-agent triciribine treatment (Figure 8B,C). Consistently, tumor tissue co-treated with triciribine and cisplatin exhibited more severe tissue damage in comparison with the single-agent cisplatin or single-agent triciribine treatment (Figure 8D).

Additionally, immunohistochemistry staining re-confirmed the high expression of ZNF275 protein in F3-PDX mice used in the present experiments (Figure 8E). The immunohistochemistry staining displayed that AKT protein expression was similar in tumor tissue of all groups (Figure 8F). The most notable inhibition of p-AKT protein expression was observed in tumor tissues treated with the combination of triciribine and cisplatin (Figure 8G).

## 4. Discussion

The therapeutic options for cervical cancer are based on the disease extent at diagnosis, clinicopathological features, risk factors, and locally available resources, which include surgery or a concurrent chemoradiotherapy [28,29]. Platinum-based chemotherapy regimens are applied clinically for advanced, metastatic, or recurrent cervical cancer, but the intrinsic or acquired cisplatin resistance seriously weakens its therapeutic efficacy in cervical cancer patients [30]. Accordingly, an intensive search for novel therapeutic modalities for cervical cancer is urgently needed. Several researchers have identified some molecular targets, such as the immune checkpoint programmed death-1 (PD-1) [31] and the tissue factor (TF) [32], associated with the development and chemo-resistance of cervical cancer; thus the targeted molecular therapy combined with chemotherapy provides new ideas for cervical cancer clinical therapy [33].

Yu et al. [34] distinguished hypoxia-immune-related prognostic differentially expressed genes in multiple myeloma patients based on the Gene Expression Omnibus database. That study indicated that ZNF275 expression was higher in high-risk multiple myeloma patients than in low-risk multiple myeloma patients [34]. Our findings illustrated that ZNF275 was highly expressed in cervical cancer tissues in contrast to surrounding normal tissues, which was consistently revealed in the GEO database. A higher expression of ZNF275 was found in the cervical cancer cell lines SiHa and HeLa in comparison with the normal cervical epithelial cell line ECT1/E6E7. Unfortunately, the present study also uncovered that ZNF275 was not highly expressed in MS751 and Caski cells, which may be due to the intrinsic cellular characteristic differences among various cervical cancer cell lines and requires further study. Additionally, our research illustrated that ZNF275 downregulation attenuated the cell viabilities, colony formation, migration, and invasion of SiHa and HeLa cells, while it enhanced cell apoptosis. However, knockdown of ZNF275 exerted no discernible effect on cell proliferation and apoptosis of ECT1/E6E7, suggesting that ZNF275 mainly functioned in cervical cancer cells.

A previous study demonstrated that depletion of ZNF217 reduced the basal phosphorylation of AKT in breast cancer cells [35]. SC79, which is an AKT agonist, has been reported to phosphorylate AKT and then modulate the biological behaviors of cells [36]. Sun et al. [37] documented that SC79 upregulated the expression of p-AKT and suppressed regorafenib-induced apoptosis, as well as restored regorafenib-mediated inhibition of proliferation in breast cancer cells, which further implicated that regorafenib exerted functions on tumor cells by deactivating the PI3K/AKT pathway. Our study revealed that the AKT/Bcl-2 pathway was attenuated by ZNF275 inhibition in SiHa and HeLa cells. Most importantly, our study demonstrated that SC79 not only reactivated the AKT/Bcl-2 pathway, but also rescued the repression of cell proliferation and reversed the induction of apoptosis in ZNF275 knockdown SiHa and HeLa cells. These findings provided further evidence that ZNF275 downregulation mediates impacts on cervical cancer cells potentially by inhibiting the AKT/Bcl-2 signaling pathway.

Littlepage et al. [38] demonstrated that the AKT signaling pathway was downstream of ZNF217, and that triciribine, which is a pharmacological inhibitor of AKT activation, attenuated the growth of breast cancer in xenografted mice models expressing high ZNF217. That study also illustrated that triciribine ameliorated the chemo-resistance of doxorubicin in breast cancer cells overexpressing ZNF217 [38]. Given our consideration of the AKT signaling pathway potentially as a therapeutically relevant target for cervical cancer cells highly expressing ZNF275, we further assessed the influence of triciribine and cisplatin on the biological behaviors of cervical cancer cells. The present study revealed that AKT and p-AKT protein were highly expressed in SiHa cells and weakly expressed in Caski cells, displaying a similar expression pattern of ZNF275 protein among different cervical cancer cell lines. We observed that AKT and p-AKT expression were reduced with the treatment of triciribine in SiHa cells expressing high ZNF275 and Caski cells expressing low ZNF275. Triciribine exerted anti-proliferative, pro-apoptotic, anti-migrative, and anti-invasive functions in SiHa cells expressing high ZNF275. Moreover, the combined treatment of triciribine and cisplatin was more effective at inhibiting proliferation, promoting apoptosis, and weakening migration and invasion of ZNF275-expressing SiHa cells than the single-agent treatment alone. Furthermore, triciribine also inhibited proliferation, migration, and invasion and promoted apoptosis of Caski cells expressing low ZNF275. Nevertheless, the combination of triciribine and cisplatin did not achieve synergistic effects on biological behaviors of Caski cells expressing low ZNF275. However, we also found that Caski cells seemed to be more sensitive to the treatment of triciribine in comparison to SiHa cells, and the underlying mechanism warrants further investigation. Our results documented that triciribine sensitized cisplatin chemotherapy efficacy in cervical cancer expressing high ZNF275.

Gloesenkamp et al. [39] documented that triciribine could act synergistically to improve the anti-proliferative efficiency of doxorubicin or 5-FU in insulinoma and gut neuroendocrine tumor cells. Another study noted that the co-treatment of triciribine and tipifarnib performed a synergistic role in suppressing anchorage-independent growth and facilitating apoptosis of breast cancer cells [40]. In addition, they also indicated that the combined therapy of triciribine and tipifarnib contributed to the significant regression of breast cancer in MMTV-Her2/*neu* Neu/ErbB2 transgenic mouse models, while the breast cancer did not respond to single-agent therapy [40]. The drug responsiveness of PDX models is highly similar to clinical chemotherapy sensitivity, because PDX models have the advantage of reproducing the genomic diversity and tumor microenvironment status of the original tumors [41]. Therefore, our study intended to evaluate the cervical cancer PDX models with a high ZNF275 protein expression in responsiveness to the combination therapy of triciribine and cisplatin. Our results revealed that triciribine exerted a tumor-inhibition function in cervical cancer PDX models highly expressing ZNF275. Moreover, the combination treatment of triciribine and cisplatin achieved synergistic anti-tumor activities and induced cervical cancer regression, which suggested that triciribine potentially sensitized cisplatin chemotherapy efficacy in ZNF275-expressing cervical cancer PDX models.

## 5. Conclusions

In this study, downregulated expression of ZNF275 showed a tumor-inhibitory impact on cervical cancer involving a weakening of the AKT/Bcl-2 signaling pathway. Moreover, triciribine, which targets and reduces signaling in the ZNF275-induced AKT pathway, exhibited a tumor-suppressive role and sensitized cisplatin chemotherapy efficacy in cervical cancer expressing high ZNF275. Our findings show that ZNF275 potentially acted as a sufficiently predictive biomarker of the therapeutic effectiveness of the combined administration of triciribine and cisplatin in cervical cancer. Taken together, combination treatment of triciribine and cisplatin greatly widened the therapeutic strategies for cervical cancer expressing high ZNF275, which sheds valuable new light on chemotherapy in combination with targeted molecular therapy for cervical cancer. Further clinical research is needed to confirm this hypothesis.

## Data Availability

The data that support the findings of this study are available from the corresponding author upon reasonable request.

## Supplementary Materials

The following supporting information can be downloaded at: https://www.mdpi.com/article/10.3390/cancers15235625/s1, Figure S1. The effect of ZNF275 knockdown on viability and apoptosis of ECT1/E6E7 cells. File S1: Western blot information.
