# Supplementary material for: The Role of ZNF275/AKT Pathway in Carcinogenesis and Cisplatin Chemosensitivity of Cervical Cancer Using Patient-Derived Xenograft Models
Source: Cancers (Basel). 2023 Nov 28;15(23):5625. doi: 10.3390/cancers15235625 (PMC10705782; doi:10.3390/cancers15235625)
Supplement: Supplementary file 1 [file cancers-15-05625-s001.zip › Figure S1.pdf]

cancers

MDPI

Article

The role of ZNF275/AKT pathway in carcinogenesis and cisplatin chemosensitivity of cervical cancer using patient-derived xenograft models

Miaomiao Ye[1,†], Tingxian Liu[1,†], Liqing Miao[1], Shuangwei Zou[1], Huihui Ji[1], Jian'an Zhang[1,*], Xueqiong Zhu[1,*]

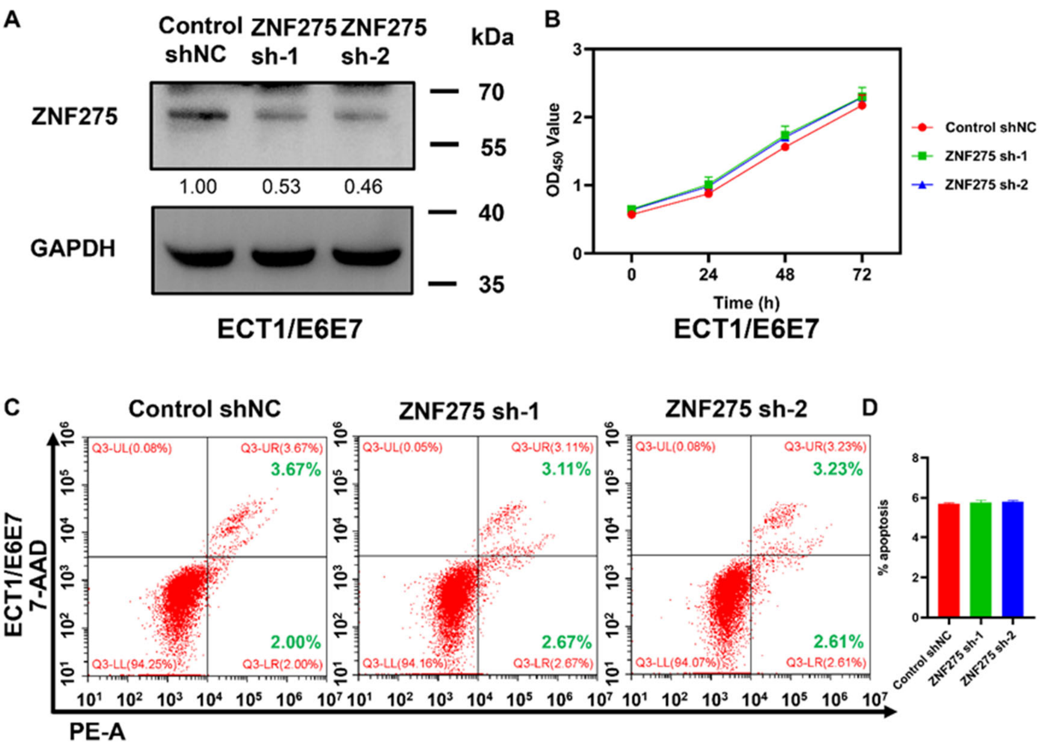

Figure S1. The effect of ZNF275 knockdown on viability and apoptosis of ECT1/E6E7 cells. A: The efficiencies of ZNF275 knockdown in ECT1/E6E7 cells were validated by Western blotting. B: CCK-8 assay revealed the effect of ZNF275 downregulation on viabilities of ECT1/E6E7 cells. C: Flow cytometric analysis revealed the effect of ZNF275 downregulation on apoptosis of ECT1/E6E7 cells. D: Quantitative analysis of the results of C.
