# Supplementary material for: The Role of ZNF275/AKT Pathway in Carcinogenesis and Cisplatin Chemosensitivity of Cervical Cancer Using Patient-Derived Xenograft Models
Source: Cancers (Basel). 2023 Nov 28;15(23):5625. doi: 10.3390/cancers15235625 (PMC10705782; doi:10.3390/cancers15235625)

Figure 1A ZNF275 protein expression in cervical cancer tissue and the adjacent normal tissue

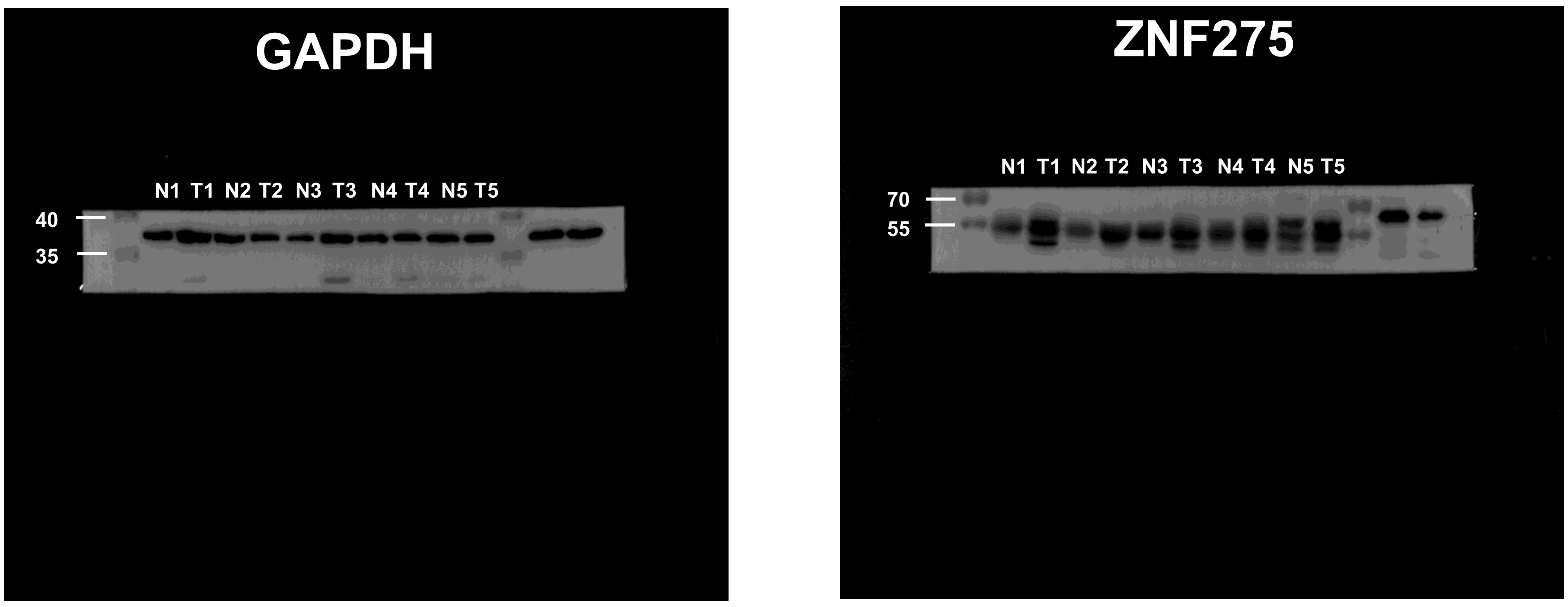

Figure 1B ZNF275 protein expression in cervical cancer cell lines.

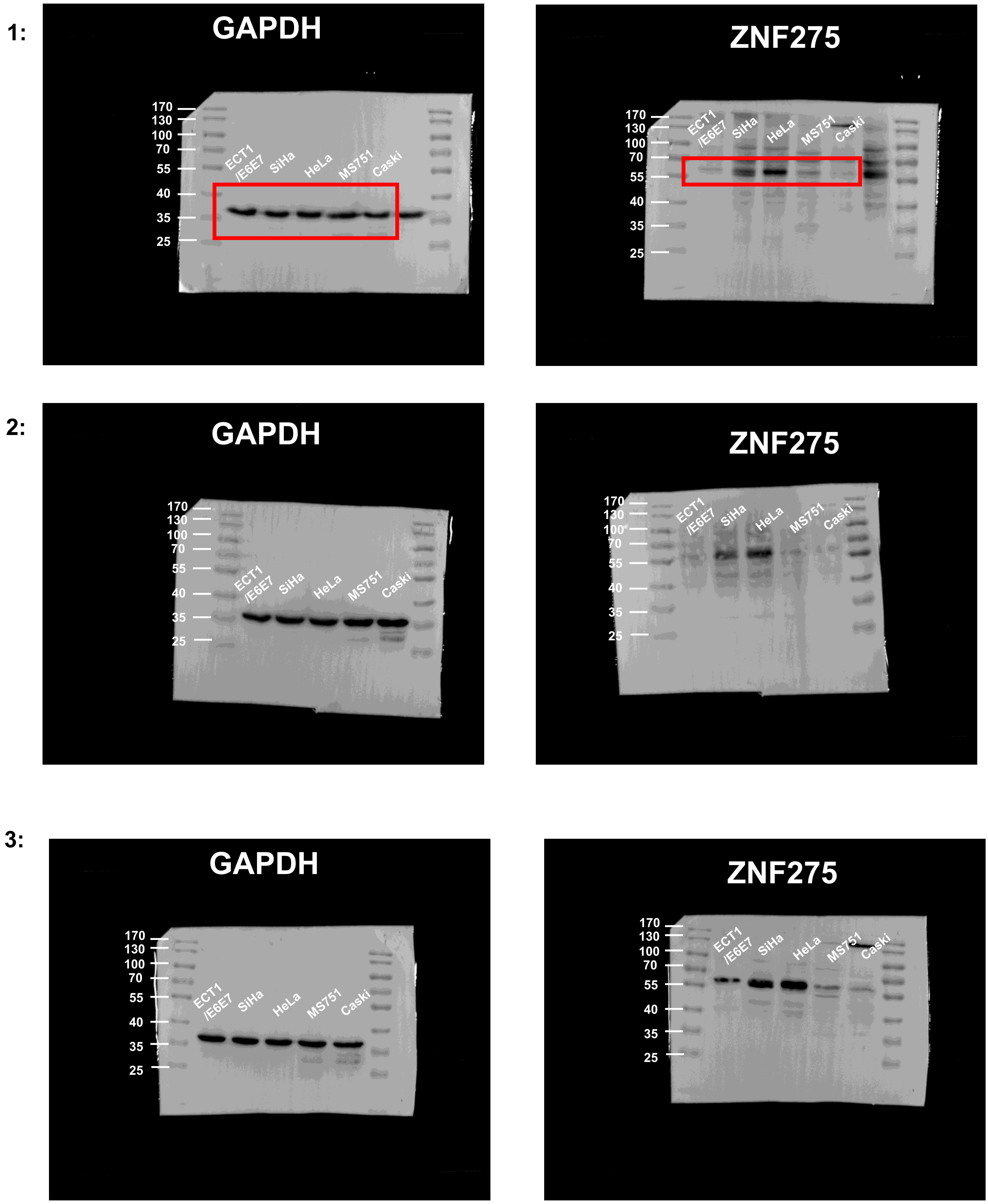

Figure 1D The efficiencies of ZNF275 knockdown in SiHa cells.

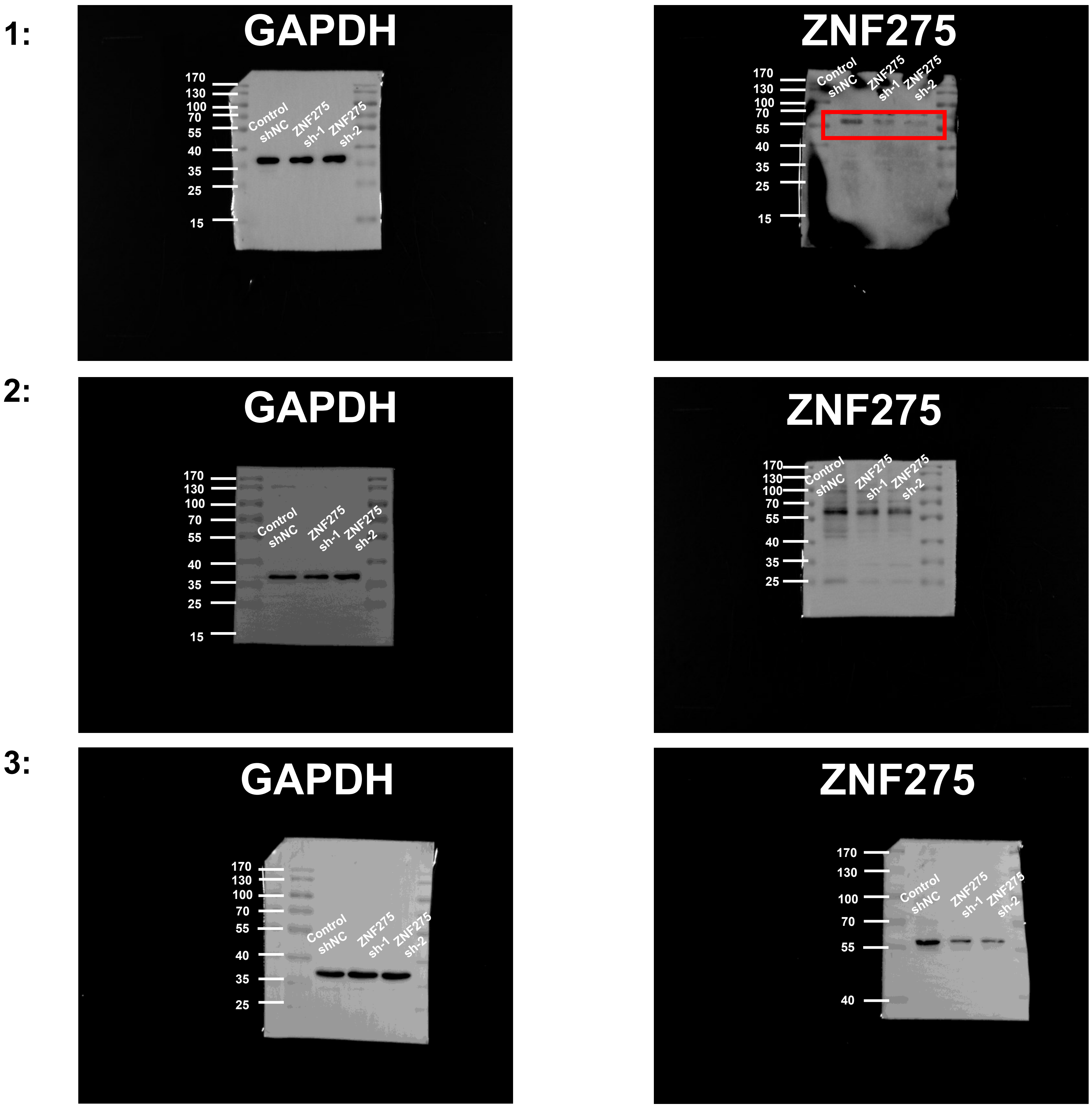

Figure 1D The efficiencies of ZNF275 knockdown in HeLa cells.

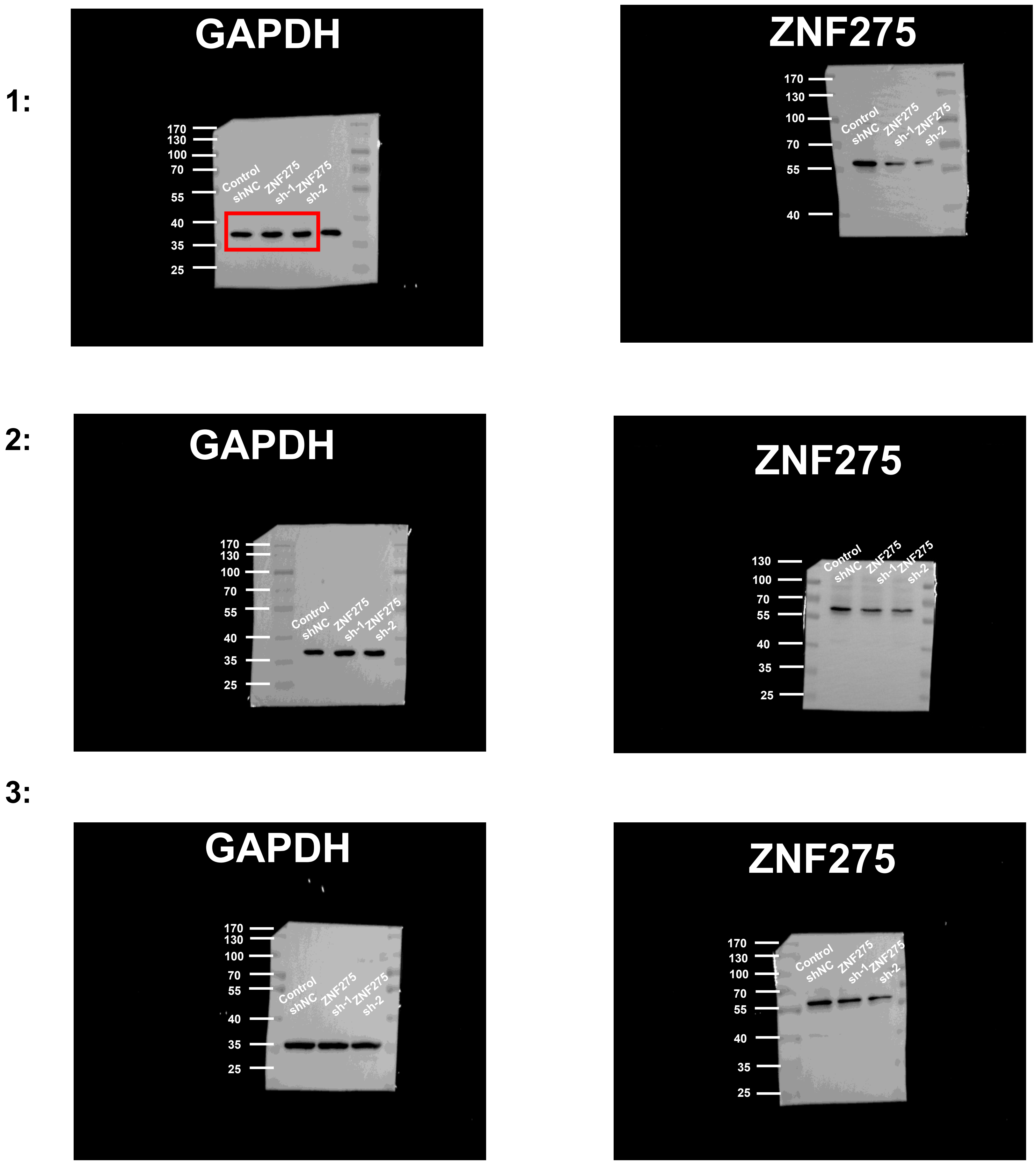

Figure 3F The expression level of AKT and p-AKT protein in ZNF275 knockdown SiHa cells in comparison of their control groups.

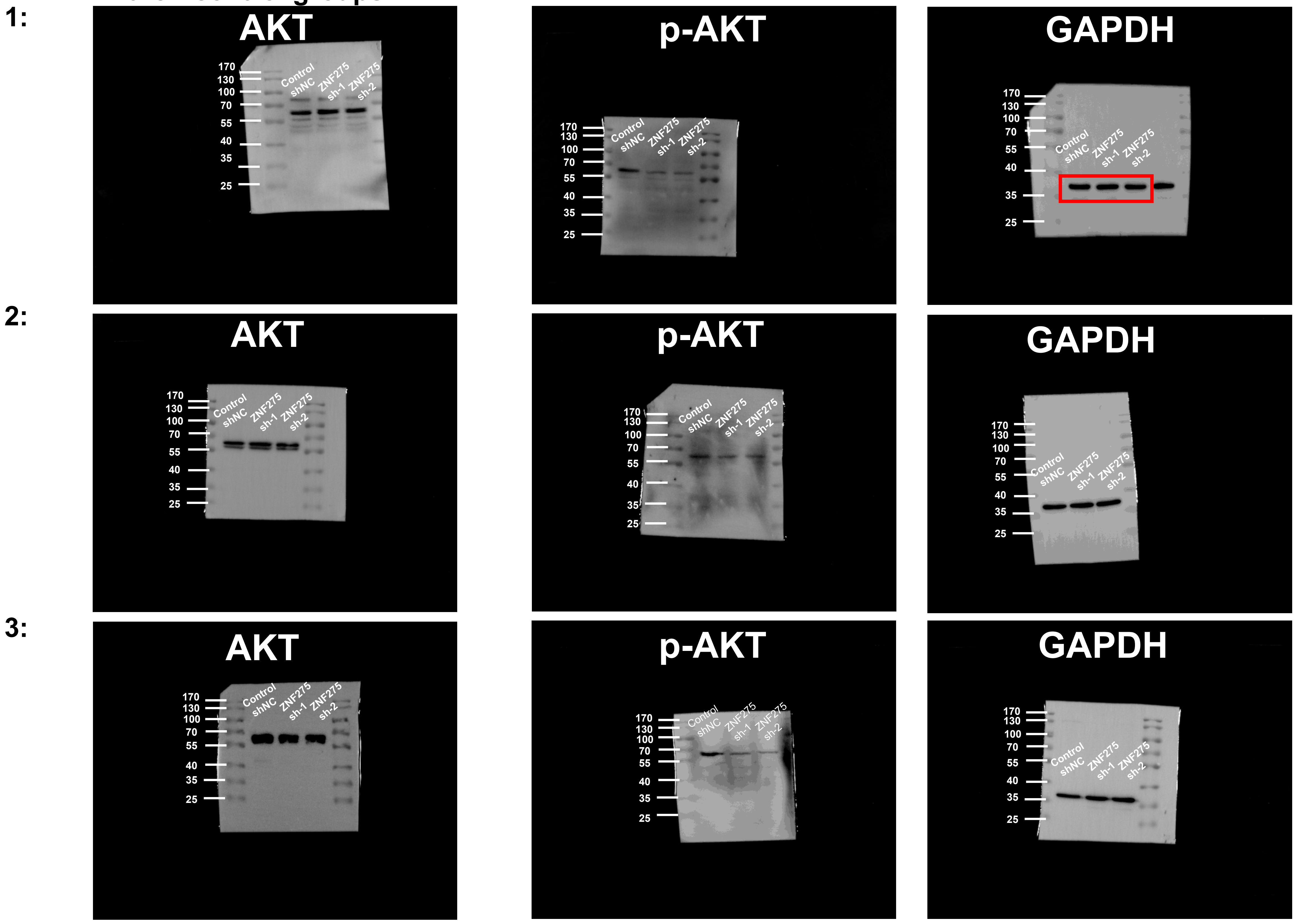

Figure 3F The expression level of AKT and p-AKT protein in ZNF275 knockdown HeLa cells in comparison of their control groups.

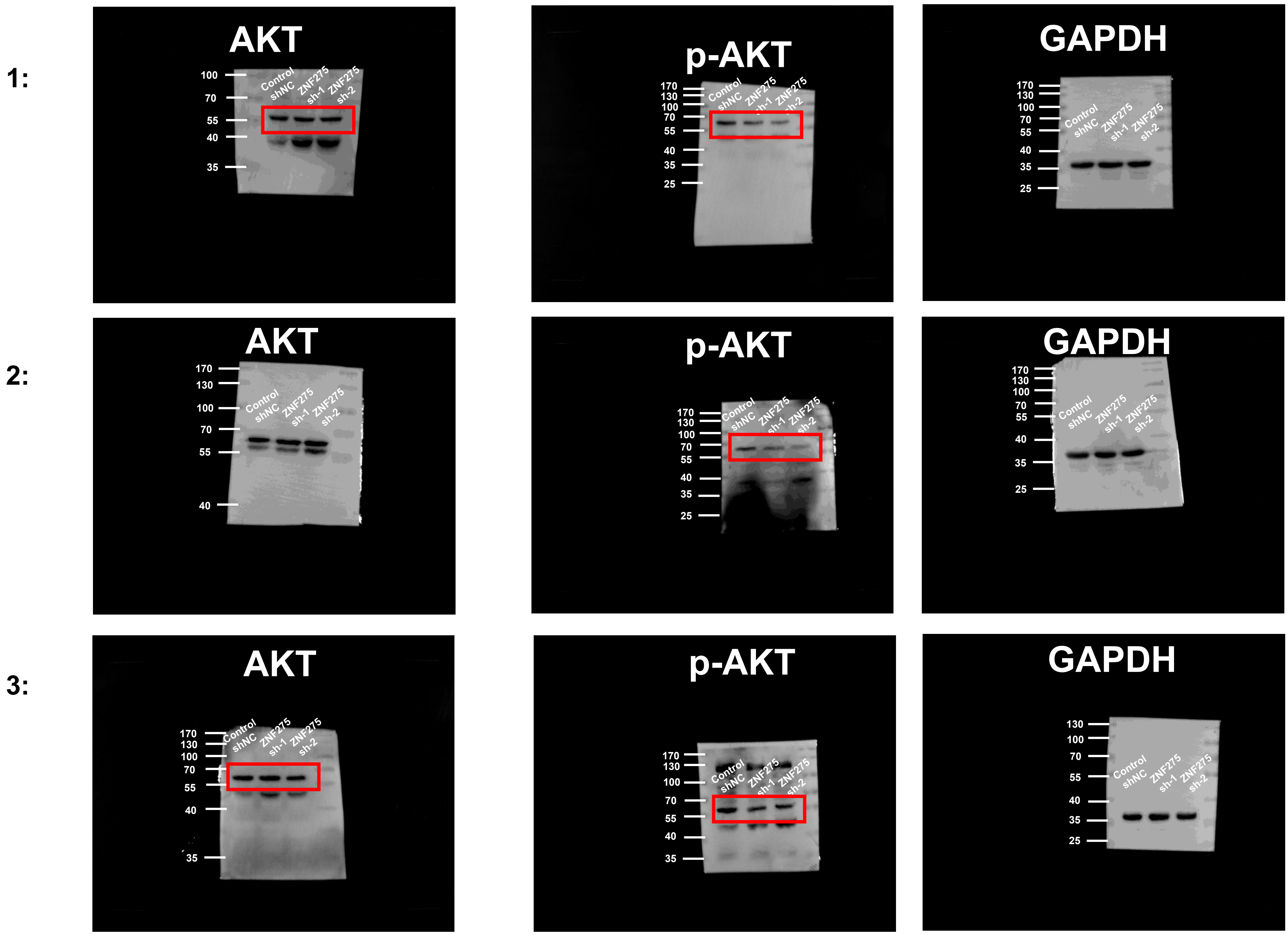

Figure 3G The expression level of apoptosis-associated protein Bcl-2 and Bax in ZNF275 knockdown SiHa cells in comparison of their control groups.

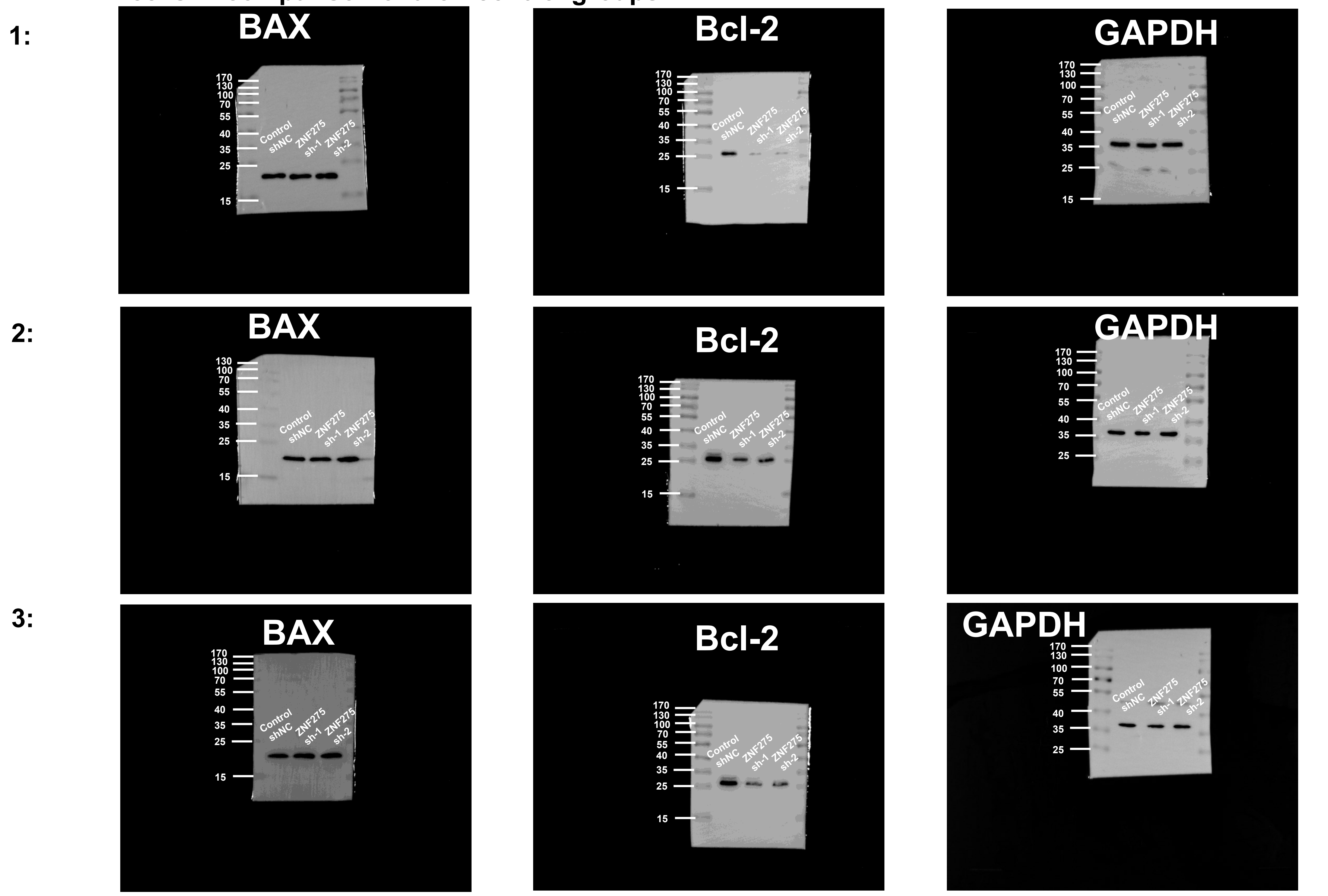

Figure 3G The expression level of apoptosis-associated protein Bcl-2 and Bax in ZNF275 knockdown HeLa cells in comparison of their control groups.

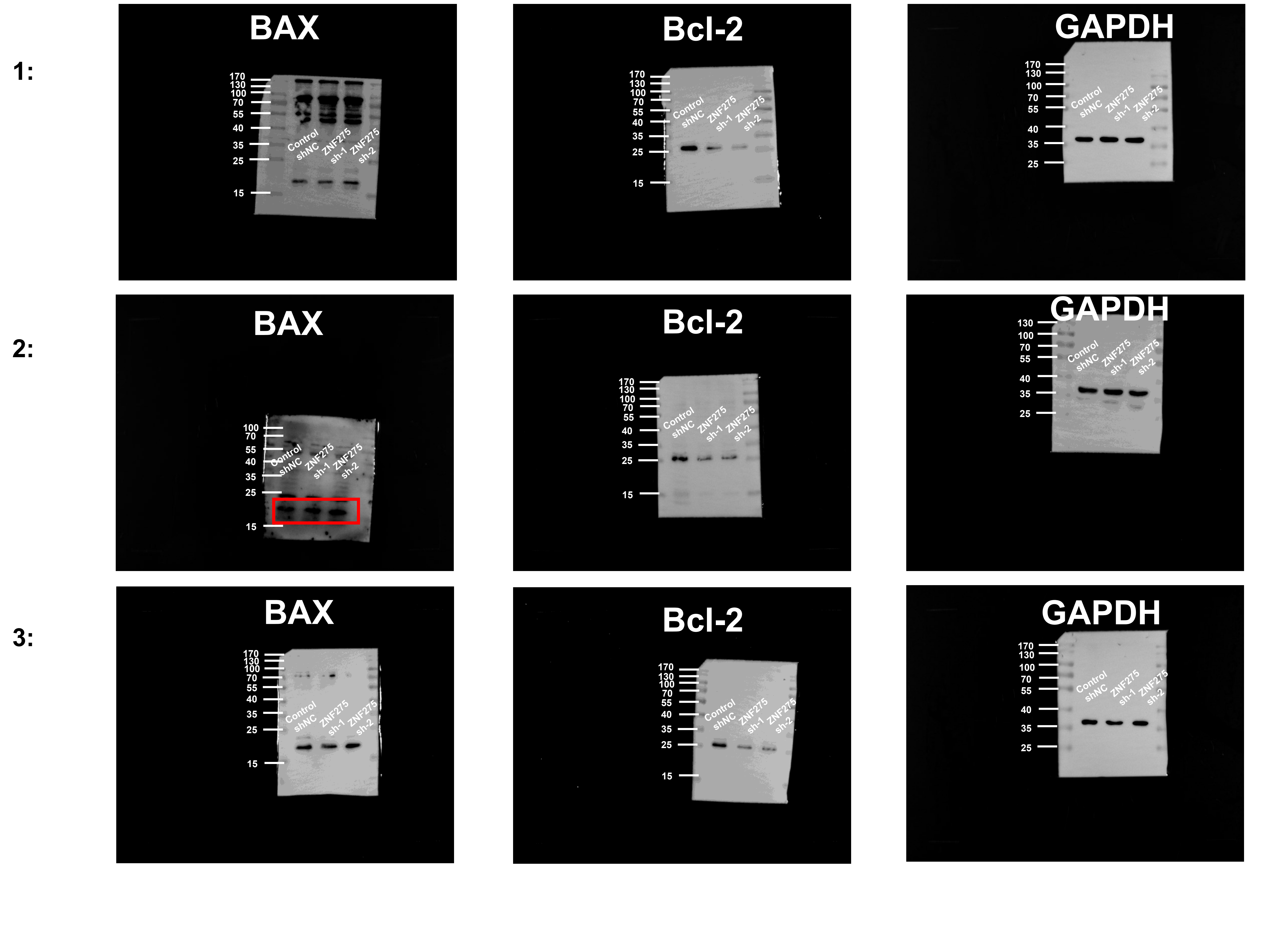

Figure 4A The expression of AKT and p-AKT protein in SiHa cells.

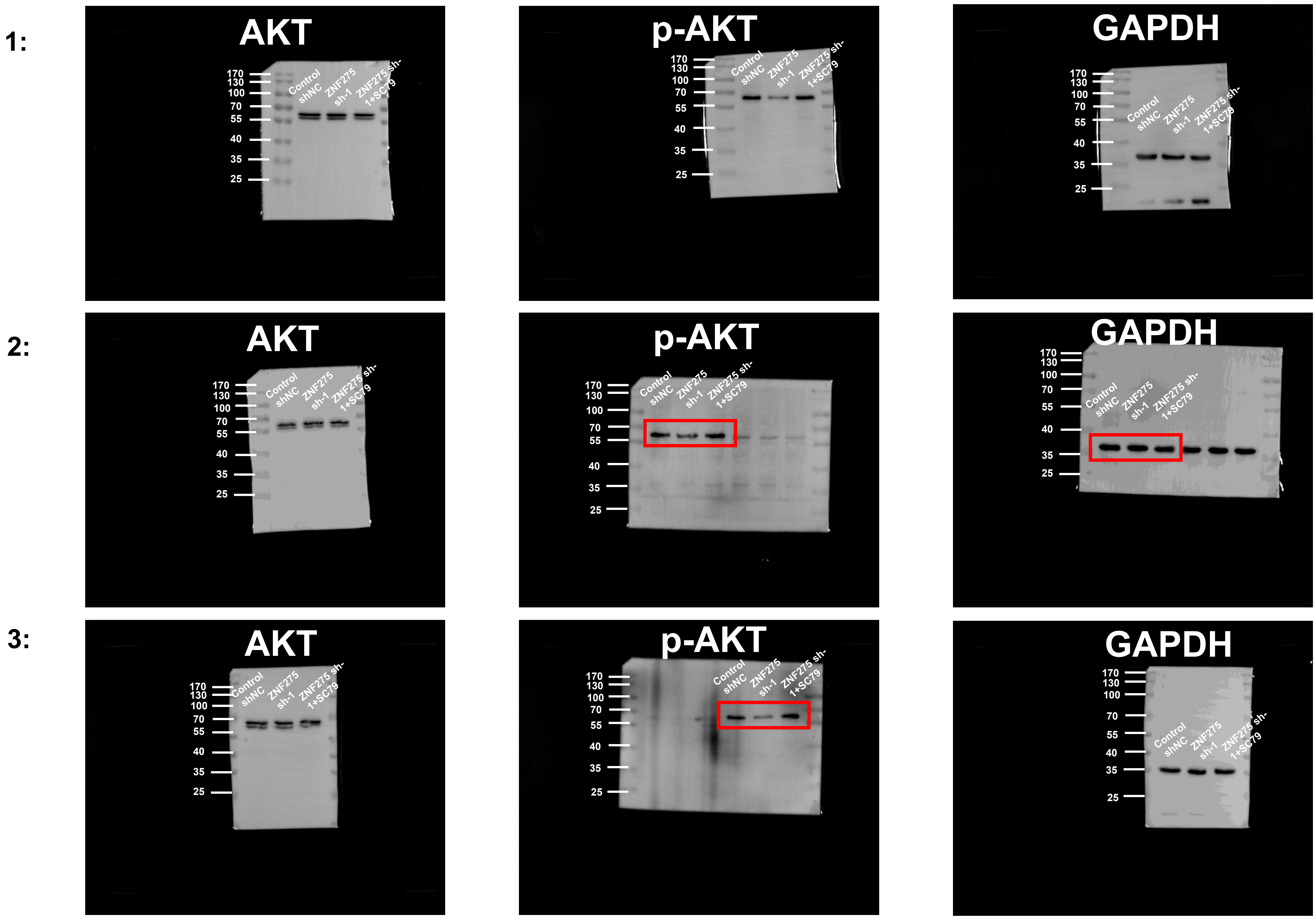

Figure 4A The expression of AKT and p-AKT protein in HeLa cells.

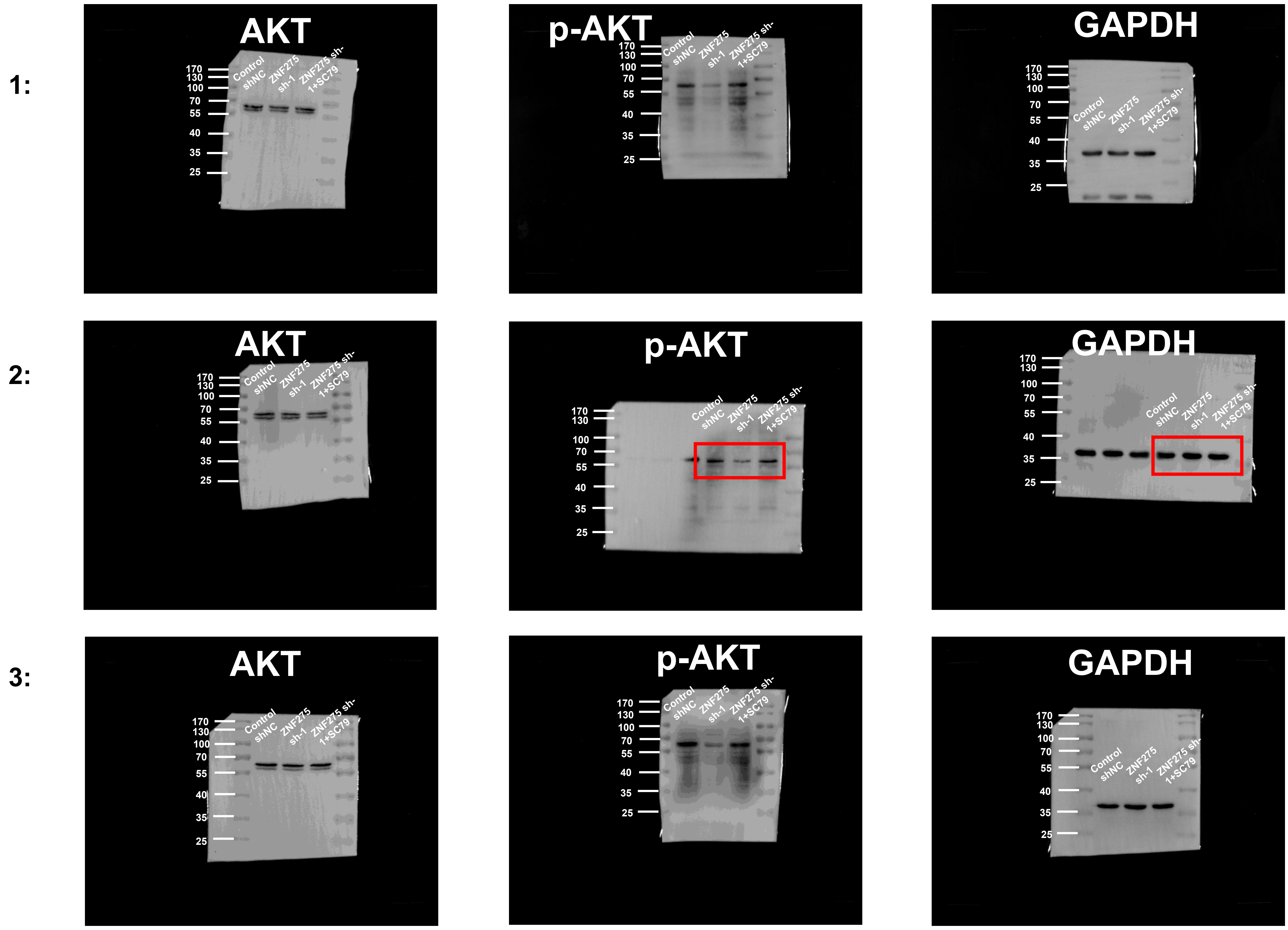

Figure 4B The expression of Bcl-2 and Bax protein in SiHa cells.

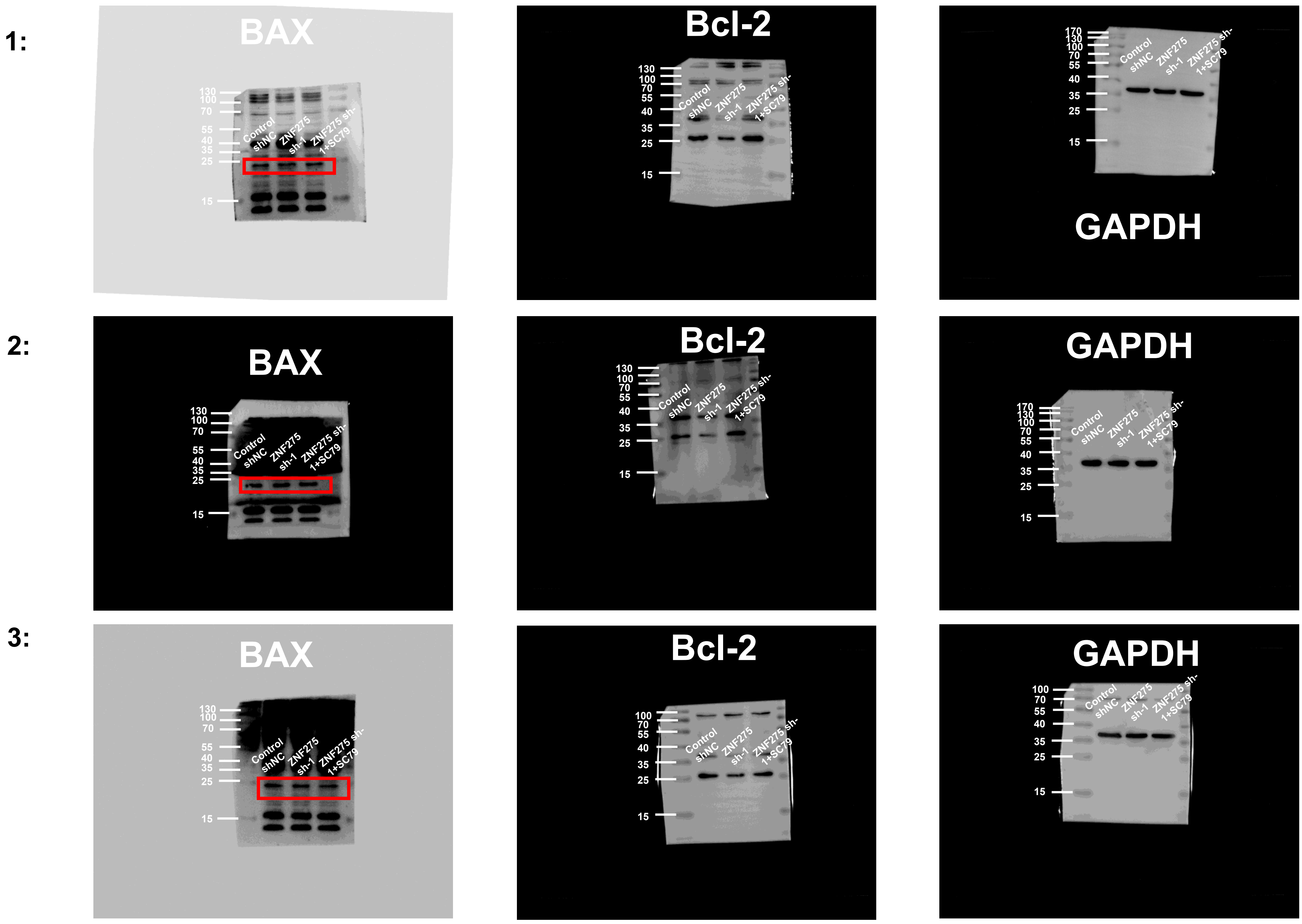

Figure 4B The expression of Bcl-2 and Bax protein in HeLa cells.

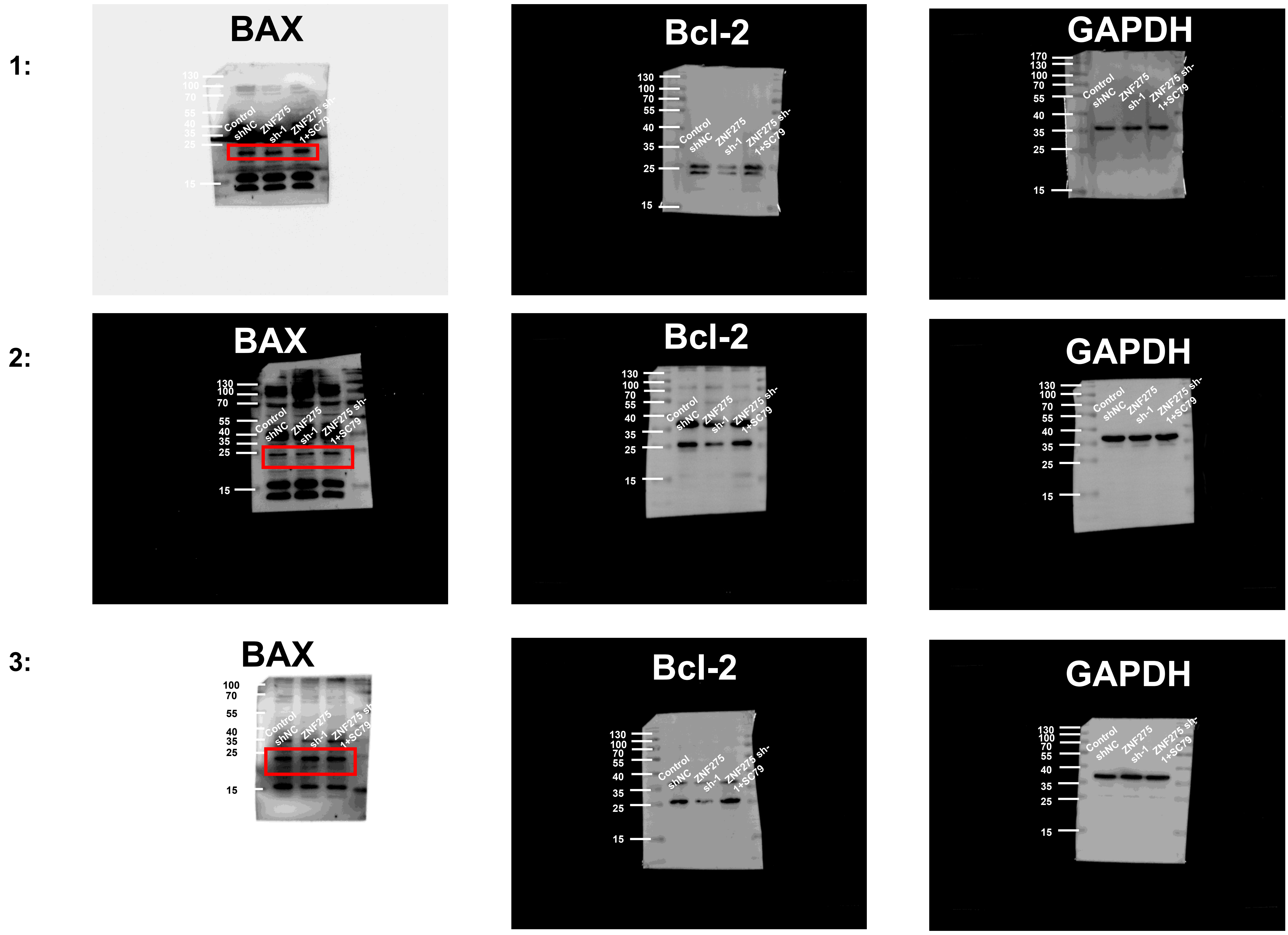

Figure 5A The expression of AKT and p-AKT protein in cervical cancer cell lines.

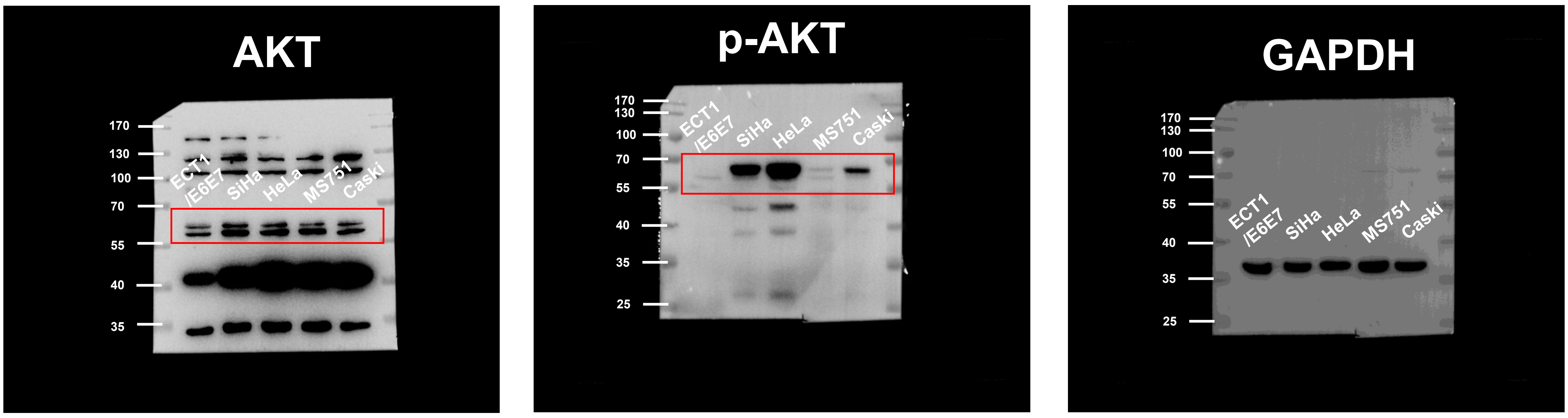

Figure 5C The effect of triciribine (100 μM for 48 h) on AKT and p-AKT protein expression in SiHa cells.

1:

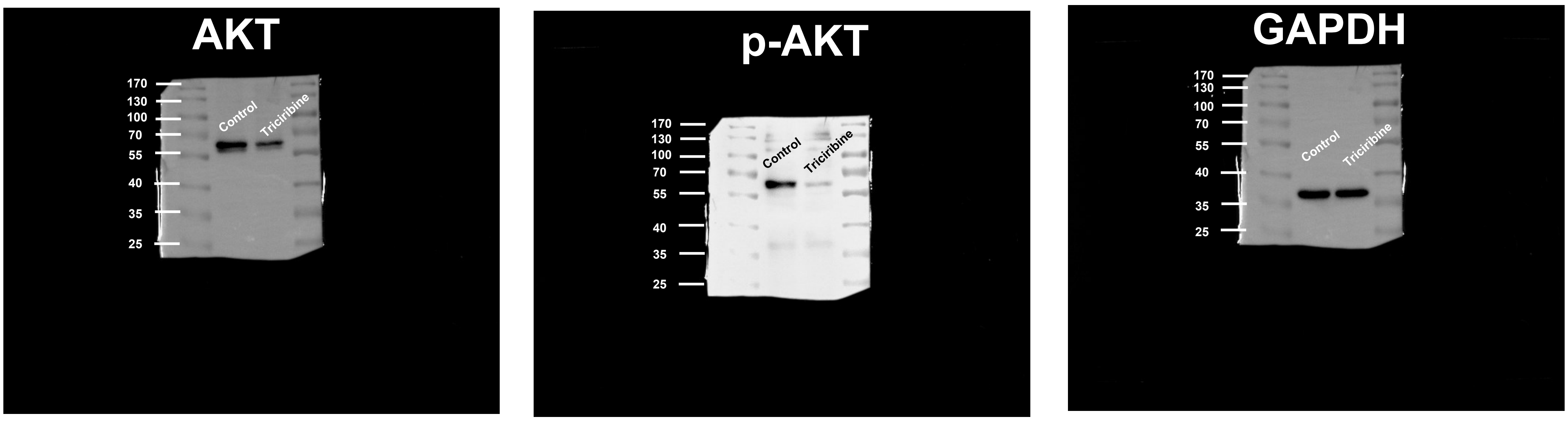

2:

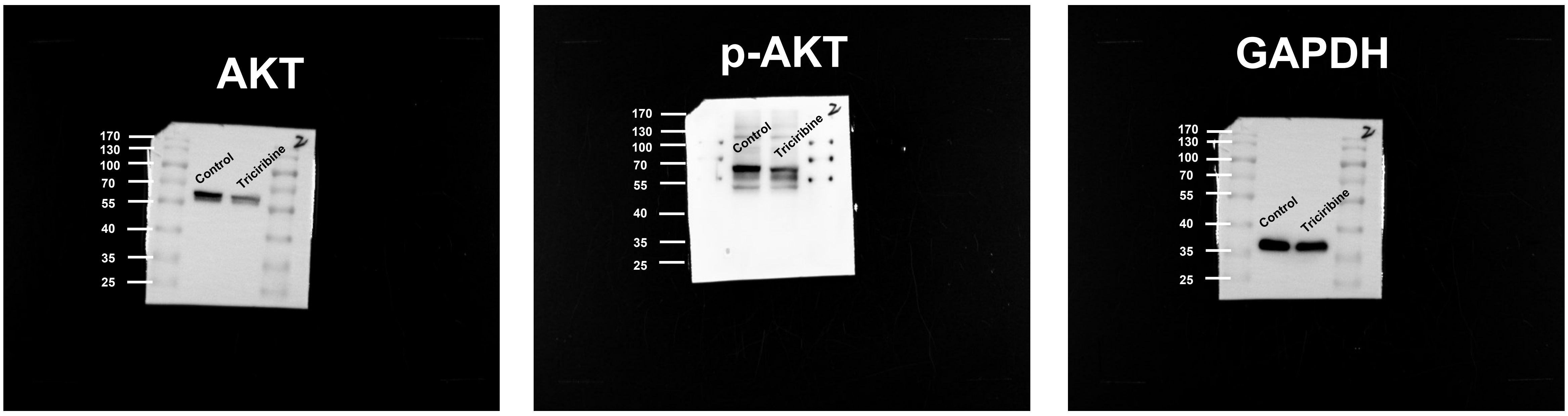

3:

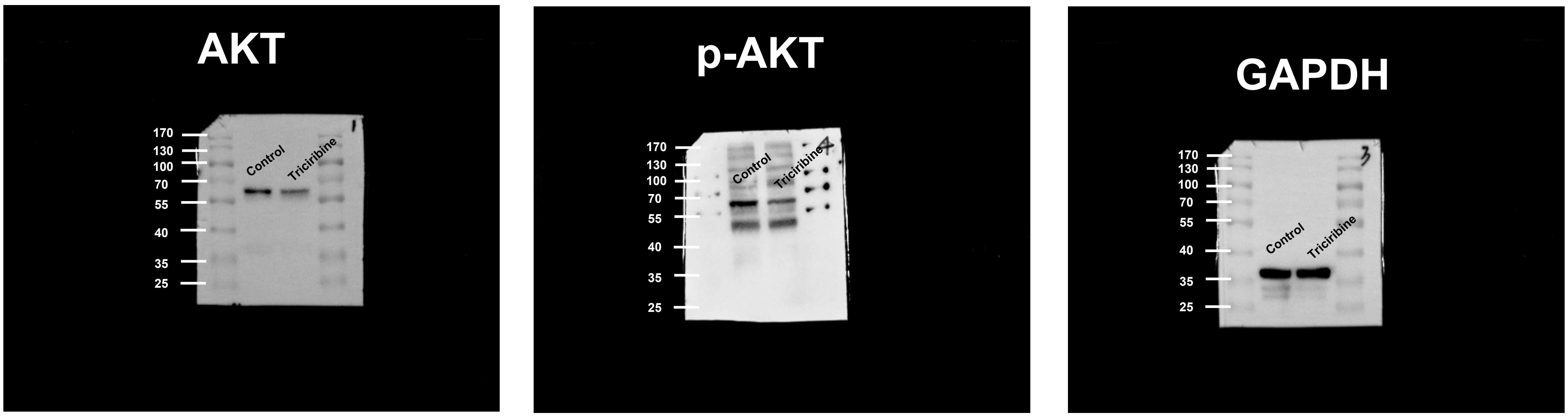

Figure 5C The effect of triciribine (12.5 μM for 48 h) on AKT and p-AKT protein expression in Caski cells

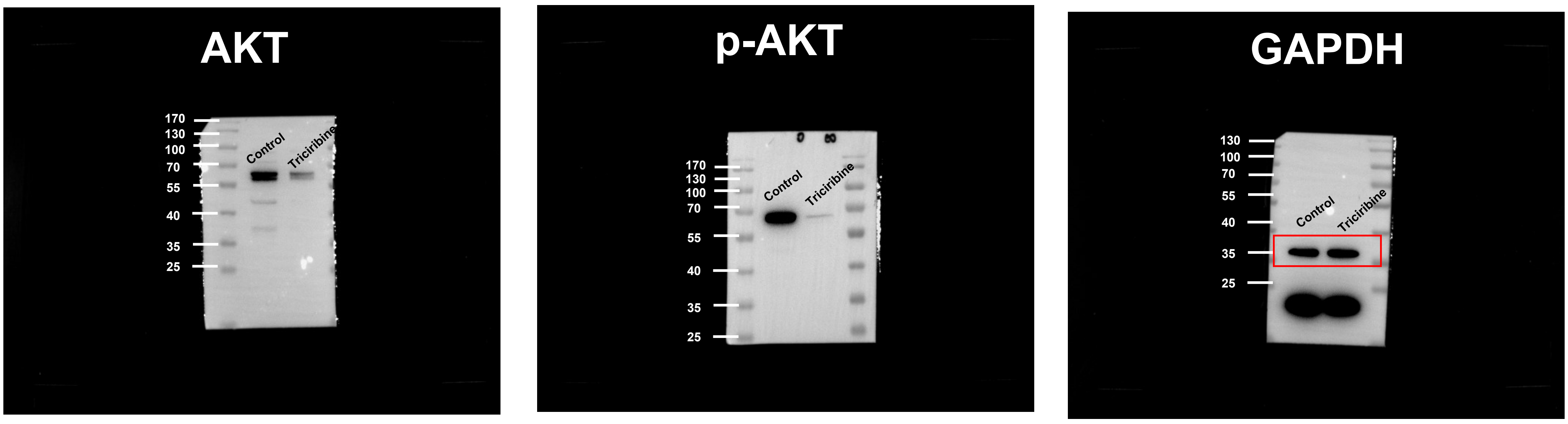

Supplementary Figure 1A The efficiencies of ZNF275 knockdown in ECT1/E6E7

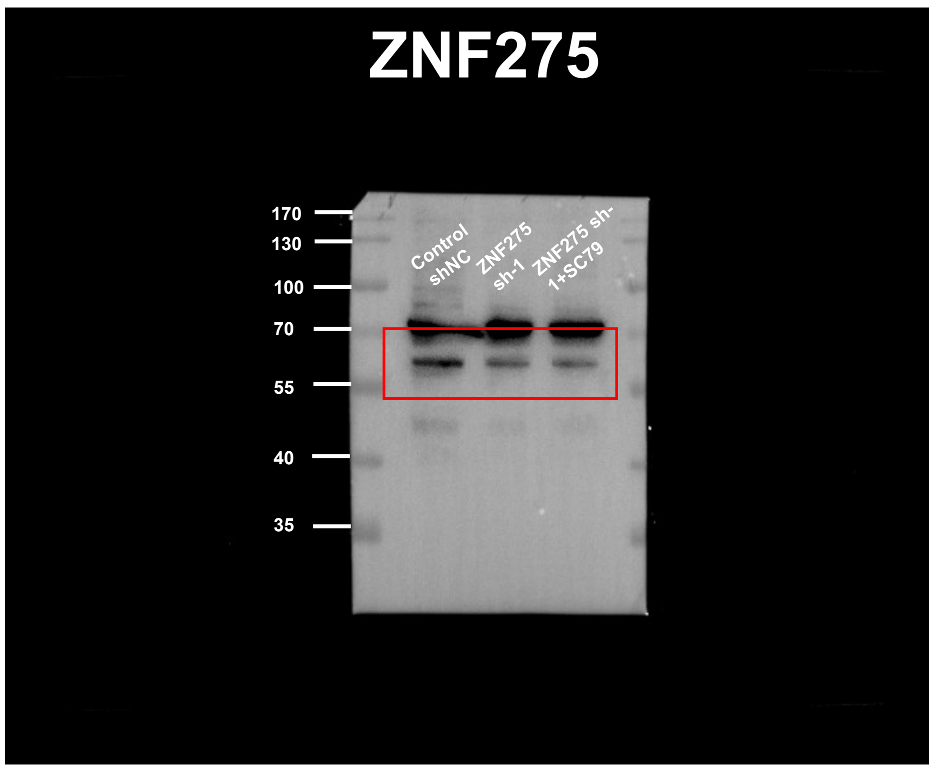

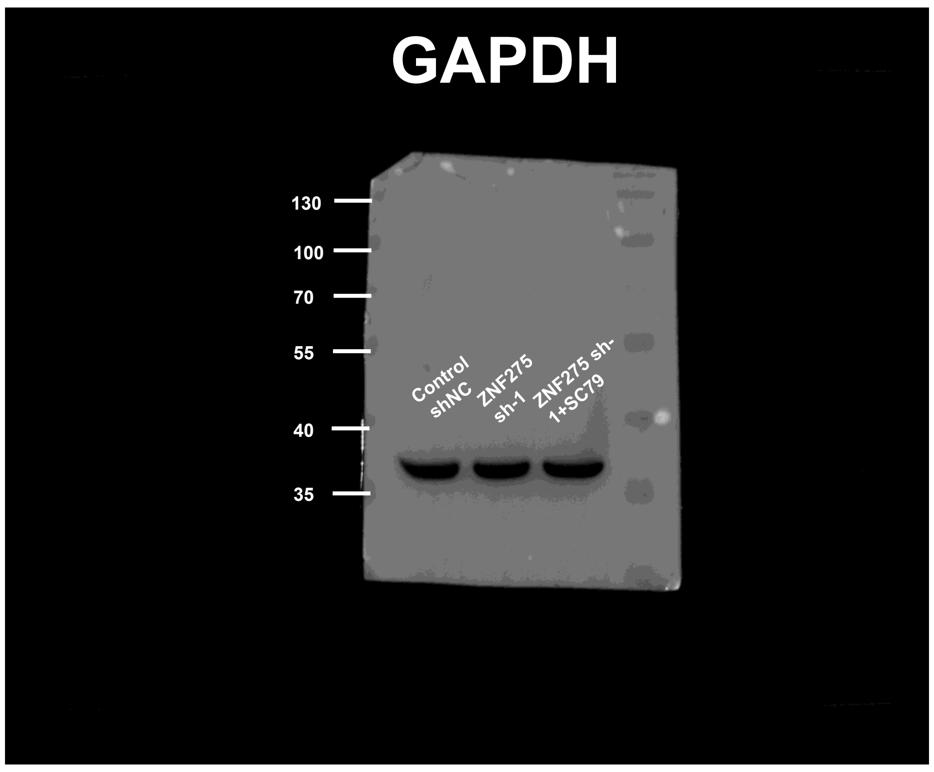

Supplement: Supplementary file 1 [file cancers-15-05625-s001.zip › File S1.pdf]
